# Engineering copper ferrite (CuFe_2_O_4_) nanosystems for medicine: a comprehensive review from design to biomedical applications

**DOI:** 10.1038/s41378-026-01383-1

**Published:** 2026-09-14

**Authors:** Maryam Malekpour, Milad Mohkam, Zeynab Karimi, Aydin Berenjian, Alireza Ebrahiminezhad

**Affiliations:** 1 Department of Medical Biotechnology, and Cellular and Molecular Research Center, Gerash University of Medical Sciences, Gerash, Iran; 2 Biotechnology Research Center, Shiraz University of Medical Sciences, Shiraz, Iran; 3 Zanjan Pharmaceutical Biotechnology Research Center, Zanjan University of Medical Sciences, Zanjan, Iran; 4 Allergy Research Center, Shiraz University of Medical Sciences, Shiraz, Iran; 5 Department of Chemical and Biological Engineering, Colorado State University, Fort Collins, CO USA

**Keywords:** Nanobiotechnology, Nanoscale materials, Nanoscale materials

## Abstract

Biomedical micro/nanosystems are rapidly advancing toward integration of therapeutic and diagnostic nanoscale agents into unified platforms. Engineers still struggle to precisely control multiple functions in biocompatible designs. Copper ferrite (CuFe_2_O_4_) nanoparticles represent a compelling material solution, combining structural versatility, magnetic responsiveness, and inherent catalytic activity within a single spinel ferrite framework. This review moves beyond a conventional materials catalog to critically analyze the engineering levers—synthesis techniques, ion doping, surface modification, and hybrid nanostructure design—that govern key physicochemical parameters and, in turn, downstream system performance. Through a comprehensive survey of the literature, we establish an engineering–property–application framework that links core characteristics (e.g., crystal structure, magnetic behavior, toxicity) to specific biomedical outcomes, including anticancer therapy, magnetic hyperthermia, targeted drug delivery, biosensing, magnetic resonance imaging (MRI) contrast enhancement, and antibacterial effects. This analysis positions CuFe_2_O_4_ nanoparticles as multifunctional components for integrable theranostic micro/nanosystems and delineates critical research gaps in long‑term in vivo safety, scalable synthesis, and clinical translation, thereby providing a foundational resource for advancing nanomedicine.

**Figure:**
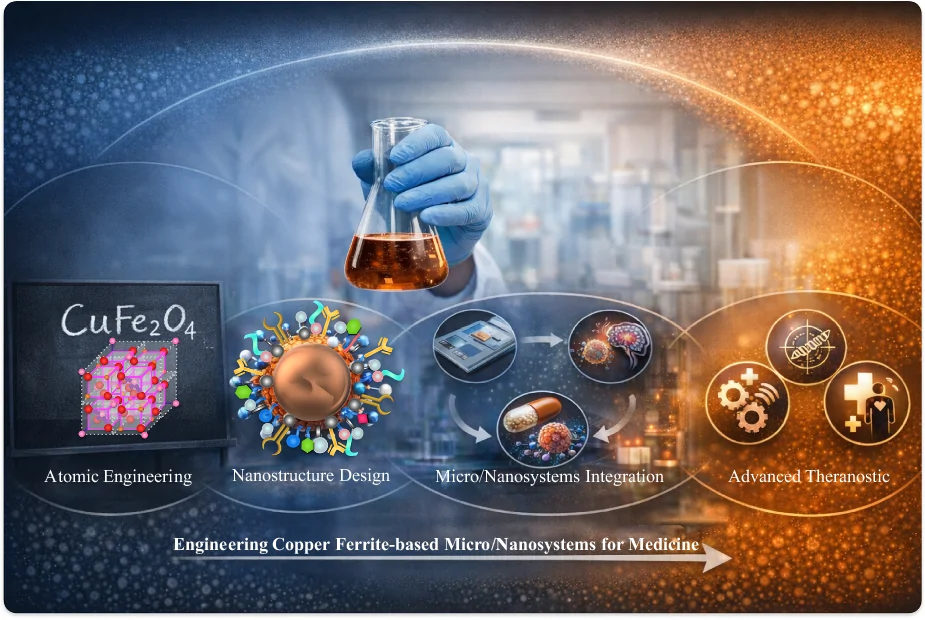

## Introduction

Biomedical micro/nanosystems increasingly incorporate nanoscale theranostic functions to achieve localized, multimodal therapy and diagnosis under clinically safe operating conditions^1,2^. Magnetic nanoparticles, in particular, have fueled major progress in biomedicine by improving diagnostics, enabling targeted therapies, and enhancing drug delivery tools^3–6^. While magnetite (Fe_3_O_4_) nanoparticles have long led the field, spinel ferrites (MFe_2_O_4_, where M = Mn, Fe, Co, Ni, Zn, or Cu) have gained significant attention for their superior physicochemical properties^7,8^.

Spinel ferrite nanoparticles feature ordered metal cations and oxygen anions in defined crystal lattices, yielding diverse structures. These nanoparticles are generally classified into soft and hard ferrites based on their magnetic behavior, specifically their response to magnetization and demagnetization^9–12^. Soft ferrites (e.g., CuFe_2_O_4_, ZnFe_2_O_4_, MnFe_2_O_4_) possess low coercivity and exhibit facile magnetization reversal. These characteristics are exceptionally advantageous for biomedical microsystems, where a reliable and responsive magnetic actuation under low, biologically safe field strengths is a critical engineering requirement^13–15^.

Copper ferrite (CuFe_2_O_4_) distinguishes itself among ferrites by combining magnetic responsiveness, structural tunability, catalytic reactivity, robust stability, and cost-effective synthesis, rendering it a highly attractive platform for integrable theranostic micro/nanosystems^16–24^. Its properties are dictated not only by the spinel framework but also by unique copper-mediated electronic and structural effects, which can be engineered for biomedical applications such as targeted drug delivery, magnetic hyperthermia, biosensing, and MRI^25–31^. Furthermore, the redox-active Cu^2+^/Cu^+^ pair contributes to Fenton-like catalytic reactions, generating cytotoxic reactive oxygen species (ROS) for antimicrobial and anticancer therapies^32,33^.

**Fig. 1:**
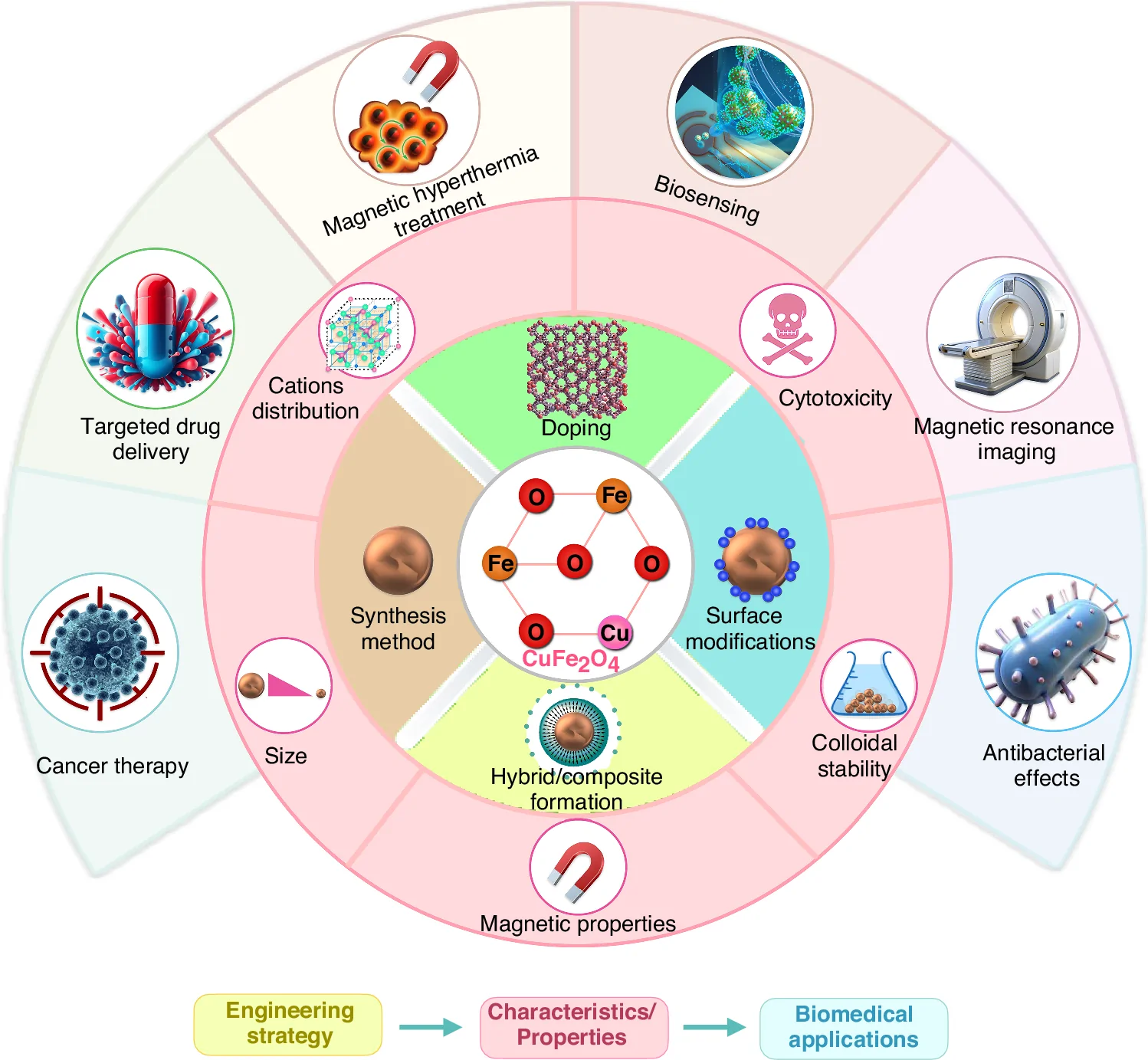
**Engineering–property–application framework for CuFe**_**2**_**O**_**4**_ **nanoparticles**. Connecting core material characteristics to targeted biomedical outcomes

Despite this potential, unmodified CuFe_2_O_4_ nanoparticles encounter clinical hurdles, including dose-dependent cytotoxicity, poor colloidal stability in physiological media, and time-dependent sedimentation that reduces effective bioavailability. These issues are closely associated with particle size, crystal structure, magnetic behavior, and surface chemistry. Precise engineering via controlled synthesis conditions, ion doping, surface functionalization, and hybrid formation can effectively mitigate these challenges, thereby unlocking the system-level performance (Fig. 1)^34,35^.

Although several reviews have addressed general spinel ferrite nanoparticles in a biomedical context, researchers have not delivered a focused, mechanism-oriented analysis of CuFe_2_O_4_ as a tailored building block for biomedical micro/nanosystems^8,9,36–38^. This review therefore, investigates how fabrication approaches, along with engineering strategies, control the physicochemical and magnetic properties of CuFe_2_O_4_ and directly determine performance across biomedical applications. We place particular emphasis on distinguishing CuFe_2_O_4_ from other ferrite systems, consolidating toxicity mitigation, and deriving design principles that enable its seamless integration into functional systems. By tackling these factors, we seek to establish CuFe_2_O_4_ as a novel, multifunctional core material for biomedical platforms, advancing nanomedicine while highlighting directions for future studies to overcome current limitations.

## Materials and methods

### Search strategy

A comprehensive literature search was performed across multiple electronic databases, including PubMed, Google Scholar, Web of Science, and EMBASE. The search was conducted from inception to October 2025 to capture the most recent publications. Key search terms included: “Ferrite nanoparticles,” “Copper ferrite nanoparticles,” “Synthesis of copper ferrite nanoparticles,” “The crystal structure of CuFe_2_O_4_,” “Surface modification of CuFe_2_O_4_,” “CuFe_2_O_4_ doping,” “Toxicity of copper ferrite nanoparticles,” “Anticancer,” “Magnetic hyperthermia therapy,” “Antioxidant,” “Drug delivery,” “Biosensor,” “MRI,” “Antibacterial,” and “CuFe_2_O_4_ in-vivo.” Boolean operators (AND/OR) were used to combine terms, and searches were limited to peer-reviewed articles in English. Additional manual searching of reference lists from relevant reviews and key articles was performed to identify any missed publications. No gray literature (e.g., conference abstracts) was included to maintain focus on high-quality, peer-reviewed sources.

### Data extraction

Data were extracted independently by two reviewers using a standardized form to minimize bias. For each eligible study, key details were recorded, including the first author’s last name, year of publication, synthesis method (e.g., co-precipitation, hydrothermal), engineering strategy (e.g., doping, hybrid/composite formation, or surface functionalization), toxicity assessment, characteristics, biomedical properties (e.g., anticancer efficacy, SAR in MHT), and advantages of the nano-formulation (e.g., biocompatibility enhancements). Data were synthesized narratively, with thematic grouping by topic (e.g., engineering, properties, applications). No quantitative meta-analysis was performed due to heterogeneity in study designs, but critical appraisal was conducted using the AMSTAR 2 tool to assess review quality where applicable.

#### Crystall structure of CuFe_2_O_4_ nanoparticles

**Fig. 2:**
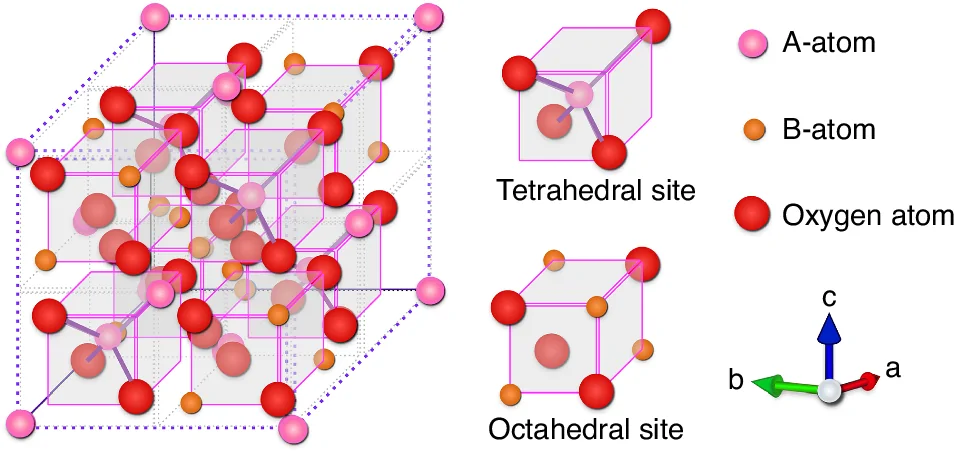
**General crystal structure of ferrites**. Schematic illustration of the general crystal structure of ferrites, showing the cubic configuration with A and B sites; The two isolated cubes in the figure are simplified models used to illustrate the local atomic environments within the spinel crystal structure

The CuFe_2_O_4_ crystal structure comprises a cubic close-packed oxygen lattice, with Cu^2+^ ions and Fe^3+^ cations occupying interstitial tetrahedral (A) and octahedral (B) sites (Fig. 2)^39^. Cation occupancy dictates spinel type: normal (Cu^2+^ at A, Fe^3+^ at B), inverse (Fe^3+^ at A, equal Cu^2+^/Fe^3+^ at B), or mixed (random occupation of cations)^40^. A defining structural feature of CuFe_2_O_4_ is the Jahn–Teller distortion induced by octahedrally coordinated Cu^2+^ ions, which elongates the c‑axis (c/a > 1), lowers symmetry from cubic to tetragonal, and enhances magnetocrystalline anisotropy and electronic localization. These changes strongly influence both magnetic behavior and catalytic performance (redox activity)^41–43^.

The inversion parameter and the resulting crystalline phase (cubic versus tetragonal) are directly tunable via synthesis conditions and thermal history, offering a practical handle to optimize properties for specific applications. In practice, tetragonal phases are often exploited where high catalytic or redox activity is desired, whereas cubic phases are favored in contexts that prioritize stronger or more predictable magnetic performance^44^.

Notably, the precise transition temperature can vary between different CuFe_2_O_4_ nanoparticle samples^45^. A neutron diffraction study by Balagurov et al.^46^ constructed a detailed thermal phase map, confirming an inverted tetragonal phase stable up to ~660 K, where a cubic phase appears. Upon further heating, the tetragonal phase is lost beyond ~700 K, with the high-temperature phase exhibiting a fully inverted cation arrangement^46^.

#### Magnetic properties of CuFe_2_O_4_ nanoparticles

**Fig. 3:**
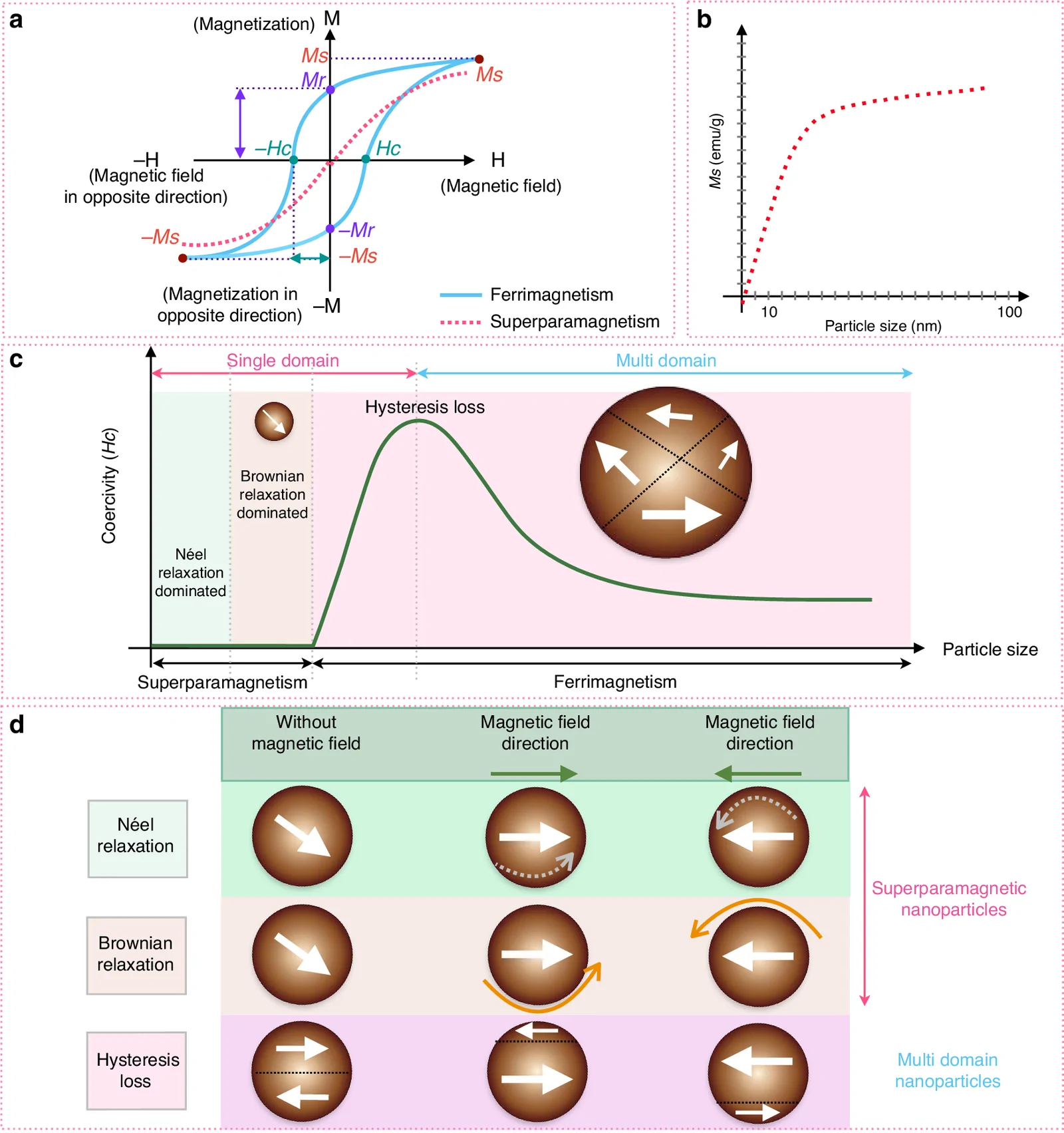
**Magnetic properties and dynamic response of CuFe**_**2**_**O**_**4**_ **nanoparticles**: **a** Hysteresis loop (Magnetization, M, vs. Applied Field, H) illustrating key parameters: Saturation magnetization (*M*_*s*_), remnant magnetization (*M*_*r*_), and coercivity (*H*_*c*_); **b** Diagram depicting the positive correlation between nanoparticle size and saturation magnetization; **c** Schematic of *H*_*c*_ evolution with particle diameter. Coercivity increases in the single-domain region, peaks at a critical size, and drops to zero in the superparamagnetic regime. Dominant heat generation mechanisms (Néel vs. Brownian relaxation) are indicated for each region, with the corresponding hysteresis loop shapes for multi-domain and single-domain particles. **d** Schematic illustrating the response to an alternating magnetic field (AMF), showing magnetic moment alignment and the dominant energy dissipation mechanisms: Néel relaxation (internal spin reorientation), Brownian relaxation (physical particle rotation), and hysteresis loss (in multi-domain particles)

For biomedical micro/nanosystems, the magnetic response of CuFe_2_O_4_ nanoparticles is a critical design variable. Performance parameters such as saturation magnetization (*M*_*s*_), coercivity (*H*_*c*_), and remnant magnetization (*M*_*r*_) (Fig. 3a) can be engineered by tuning particle size, crystalline phase, and cation distribution. *M*_*s*_ represents the maximum achievable magnetization under a saturating external field, *H*_*c*_ is the field strength required to demagnetize the material, and *M*_*r*_ is the magnetization retained after field removal. All three parameters are thermally dependent, typically increasing at lower temperatures as reduced thermal fluctuations stabilize magnetic domains^47,48^.

*M*_*s*_ generally increases with crystallite size due to a lower surface-to-volume ratio, which minimizes disordered surface spins and enhances internal magnetic alignment (Fig. 3b)^49^. Nanoparticles below a critical size (~10–20 nm) or those with highly disordered phases can exhibit superparamagnetism, which is particularly advantageous in vivo. In this state, each nanoparticle acts as a single magnetic domain with a large net moment that responds to an external field but retains no magnetization after its removal, preventing aggregation (Fig. 3c)^47,48,50^.

Size‑dependent magnetic performance of the nanoparticles is fundamentally governed by magnetic relaxation dynamics, which arise from hysteresis losses, Brownian relaxation, and Néel relaxation. Hysteresis loss occurs in large/multi-domain nanoparticles due to the lag between the magnetization response of the nanoparticles and the externally applied magnetic field. Brownian relaxation involves the physical rotation of the entire nanoparticle within the surrounding fluid. Néel relaxation, on the other hand, is the internal reorientation of the magnetic moment within a fixed particle lattice, a mechanism that is predominant in small, superparamagnetic nanoparticles and is highly sensitive to the particle’s magnetic anisotropy and the field strength (Fig. 3d)^51–56^.

CuFe_2_O_4_ may exhibit ferrimagnetic behavior (up to the temperatures of 700–780 K) due to the antiparallel alignment of magnetic moments between cations occupying A and B sites in the spinel lattice, driven predominantly by strong oxygen-mediated A–B superexchange interactions^34,46,57,58^. This material occupies a beneficial intermediate magnetic regime, offering moderate coercivity and a widely tunable *M*_*s*_ (typically 20–80 emu/g) with thermally adjustable anisotropy^27,59,60^. The cubic phase of CuFe_2_O_4_ typically yields higher *M*_*s*_ and lower *H*_*c*_, favoring strong, soft magnetism^61^. As the degree of inversion increases, exchange interactions within the B sublattice intensify, which can enhance *H*_*c*_ and *M*_*r*_ but may reduce the net magnetization and *M*_*s*_ because of increased noncollinear (canted) spin arrangements^23,62–64^.

#### Synthesis of CuFe_2_O_4_ nanoparticles

**Fig. 4:**
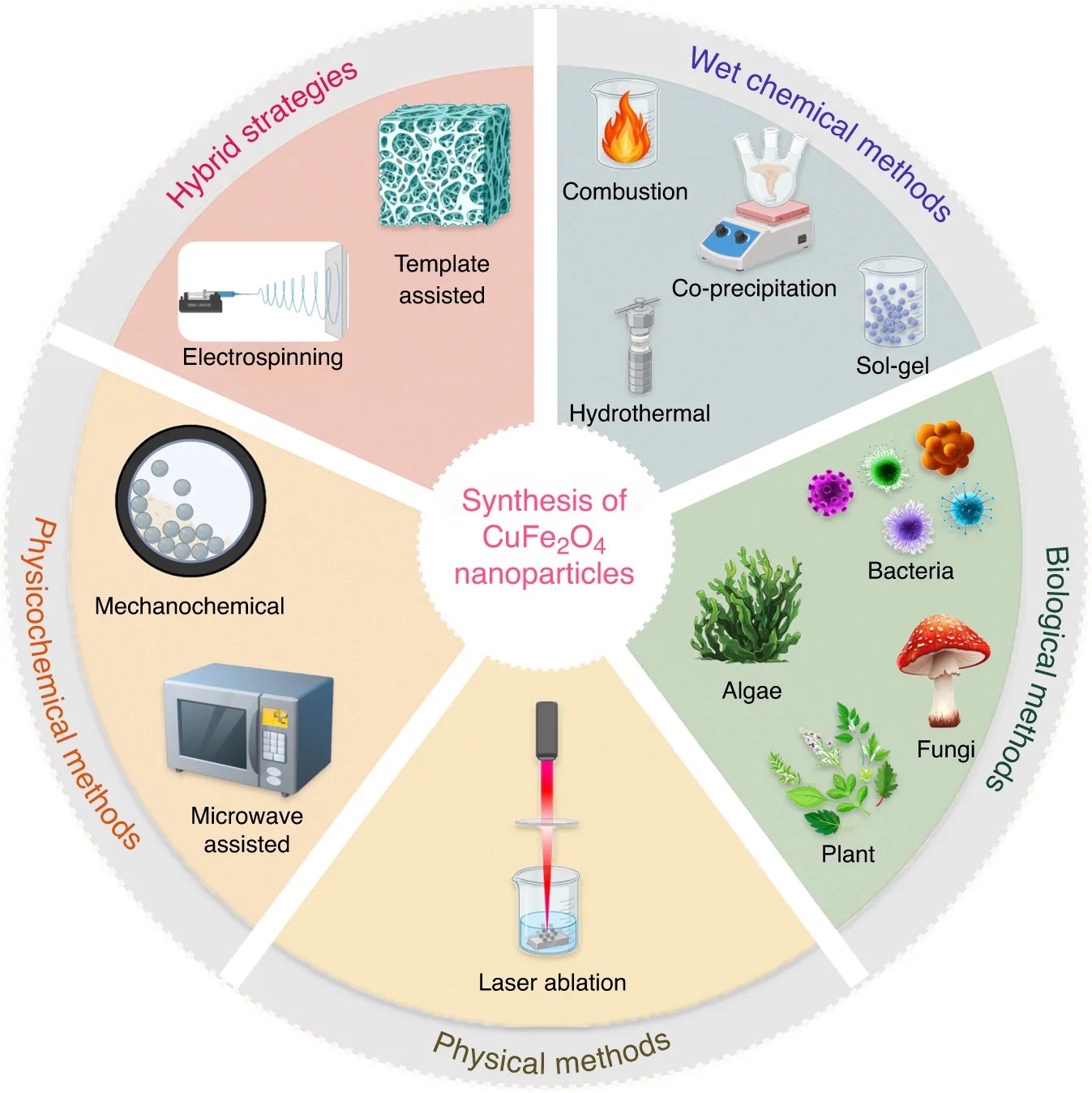
**Different developed methods for the synthesis of CuFe**_**2**_**O**_**4**_ **nanoparticles**. The available categorized approaches include wet chemical methods, biological methods, physical methods, physicochemical methods, and hybrid strategies

Designing functional platforms based on CuFe_2_O_4_ starts at the synthesis stage, where the choice of fabrication route directly determines particle size, morphology, and cation distribution. CuFe_2_O_4_ nanoparticles were produced using wet-chemical, physical, physicochemical, biological, and hybrid approaches (Fig. 4), each operating under distinct reaction conditions and offering specific advantages and limitations for biomedical integration (Table 1)^40,42,44,65,66^.

**Table 1:** Comparative summary of CuFe_2_O_4_ nanoparticles synthesis methods: Advantages, limitations, and relevance to biomedical micro/nanosystem engineering

Synthesis method	Advantages	Disadvantages	Applications in biomedical micro/nanosystem engineering	Ref.
Coprecipitation	• Simple and rapid • Aqueous-based • Low-cost • High yield • Straightforward doping • Scalable	• Aggregated nanoparticles • Broad size distribution • Poor control over size & morphology • Requirment of precise pH & temperature control	• Useful for high-volume and low-cost precursor material for composite scaffolds or where extreme monodispersed nanoparticles are not required • Requirment of post-annealing • Easy translation to GMP	^44,77^
Hydrothermal/solvothermal	• High control over crystallinity & morphology • Good purity	• Autoclave required • High/moderate energy demand • Long reaction times • Safety concerns • Moderate scalability	• Excellent for producing high-quality and application-tuned nanoparticles (e.g., nanoparticles for hyperthermia and MRI) • Useful for modifying the surface • Sealed reactor enables tailored growth of cubes, rods, and spheres	^67,68,132^
Sol–gel	• Excellent stoichiometric control • High homogeneity • Low processing temperature • Versatile doping	• Aggregated nanoparticles • High calcination temperature demand for crystallization • Requirment of solvent removal • Multi-step reaction • Long processing time	• Best for creating nanoparticle-polymer composites, coated implants, or porous scaffolds where a gel precursor is advantageous • Calcination step can limit based on bio-functionalization	^69,133^
Combustion	• Extremely rapid • Energy-efficient • Simple setup • High yield	• Exothermic reaction uses nitrates/fuel • Poor control over size & morphology	• Useful for magnetic material production • High crystallinity but often porous/agglomerated nanoparticles • Limited for biomedical integration due to poor control and agglomeration, unless followed by extensive milling/processing	^59^
Microemulsion	• Fine control over size • Synthesis of very small and uniform nanoparticles at low temperature	• High-cost • Complex purification • Very low yield • Difficult to scale	• Useful for synthesizing very small, uniform seed particles or studying fundamental size-dependent properties • Requirment of large volumes of surfactants/organic solvents	^71,134^
Polyol	• Good control over morphology • High boiling point polyol • Good crystallinity without calcination	• High temperature demand • High-cost reagents • Organic solvents • Moderate yield	• Reduction of metal salts to metal particles by poly alcohols (ethylene glycol, diethylene glycol) • Produces readily dispersible nanoparticles in organics for further polymer composite work	^70^
Green	• Eco-friendly • Renewable resources • Cost-effective • Low energy • Inherent bio-compatibility	• Sophisticated and slow • Extremely poor reproducibility & control • Low yield • Challenging scalability (batch variability)	• Useful for applications in medicine and biology, based on its inherent bio-compatibility • Promising for reducing cytotoxic byproducts • Lack of precise engineering control is a major barrier for reproducible device • Combination with other methods (e.g., coprecipitation, hydrothermal, combustion)	^72,135^
Laser ablation	• Eco-friendly • Aqueous-based • Good purity • No chemical precursors (minimal byproducts)	• Requirment of specialized equipment (high cost) • Broad size distribution • Very low yield • Limited scalability	• Useful for generating ultra-pure, ligand-free nanoparticles from bulk target • Suitable for fundamental studies on surface reactivity • Not viable for mass production of engineered systems	^24,136^
Microwave-assisted synthesis	• Rapid • Uniform heating • Energy-efficient • Good control over size	• Requirment of specialized equipment • Requirment of microwave-absorbing precursors	• A method for rapid and reproducible lab-scale synthesis • Useful for modifying the surface • Suitable for optimizing synthesis parameters for system components • Combination with other methods (e.g., combustion, sol–gel)	^18,85^
Ball milling	• Top-down, solid-state, and direct • Simple • No chemical precursors	• Requirment of specialized equipment • High energy demand • High noise and dust • Broad size distribution • Difficult to achieve <50 nm nanoparticles	• Almost exclusively for producing coarse magnetic powders • Useful for composite bulk materials and magnetic separation supports • Scalable for alloys/composites • Unsuitable for precision nanomedicine requiring small or uniform nanoparticles	^137^
Template-assisted	• Precise control over size & morphology • Excellent for ordered arrays	• Complex multi-step process • Requirment of template removal (often harsh chemicals) • Very low yield • Not scalable	• A powerful research tool for studying morphology-dependent properties (e.g., magnetic anisotropy in nanorods) • Unsuitable for therapeutic mass production but informative for design principles • Combination with other methods	^75,138^
Electrospinning	• Direct fabrication of nanofibers • Integrates nanoparticles into polymer fibers in one step	• Requirment of specialized equipment • Requirment of polymer optimization	• Suitable for creating functional high-surface-area 3D scaffolds or sensor patches where nanoparticles are a fixed component within a macroscopic device	^27,76^

Wet chemical approaches include coprecipitation, hydrothermal, solvothermal, sol–gel, combustion, microemulsion, and polyol methods. In general, the wet chemical approach typically involves assembling CuFe_2_O_4_ nanoparticles from Cu^2+^ and Fe^3+^ ions, enabling precise control over particle size and morphology. For instance, coprecipitation entails mixing cupric and ferric salts in a basic medium to precipitate CuFe_2_O_4_ nanoparticles directly^44^. Hydrothermal and solvothermal syntheses utilize a Teflon-lined autoclave to heat the reaction mixture (in water or an organic solvent) under elevated pressures and produce highly crystalline nanoparticles from metal ion precursors^67,68^. The sol–gel process includes the formation of a colloidal suspension (sol) from a heated mixture of metal ions (from metal alkoxides or metal salts) and alcohol in the presence of water. Gelation occurs as the sol progressively evolves into a gel-like network^69^. The combustion method relies on an exothermic redox reaction between a fuel and an oxidizer to rapidly generate particles from metal salts^59^. Cupric and ferric ions can also be transformed to CuFe_2_O_4_ nanoparticles through the polyol method. In this method, metal ions are dissolved in a high-boiling-point polyalcohol solvent and heat threated to form nanoparticles^70^. Microemulsion techniques confine precipitation reaction within nanodroplets, facilitating uniform particle formation from precursors^71^.

Physical approach, which follows a top-down approach, is frequently executed via laser ablation. This technique involves exposing bulk CuFe_2_O_4_ material to laser irradiation within a liquid or gaseous medium. The interaction results in the vaporization of the material, which then condenses to form nanoparticles with high purity^24^.

Physicochemical approaches utilize physical treatments to force drive chemical transformations of metal precursors to nanoparticles. Typically, these physical forces are delivered through devices such as ball mills in mechanochemical processes or microwave ovens in microwave-assisted synthesis. The mechanochemical approach employs high-energy ball milling to facilitate atomic rearrangement, whereas microwave-assisted synthesis utilizes microwave radiation to promote the formation of nanoparticles^18,49^.

Biological methods offer eco-friendly synthesis of CuFe_2_O_4_ nanoparticles by using biomolecules as stabilizer or precipitating agent that drives hydrolysis of metal precursors to nanoparticles^72–74^. Hybrid synthesis strategies integrate the advantages of multiple fabrication techniques to create nanoparticles with tailored properties for advanced biomedical applications. These methods can be implemented through template‑assisted, microwave‑assisted, or electrospinning‑based approaches, as well as other innovative hybrid protocols^31^. In template‑assisted synthesis, a physical or chemical scaffold (such as a porous membrane, polymer matrix, or fibrous framework) is used to precisely control nanoparticle morphology, size, and spatial arrangement^75^. The electrospinning technique utilizes electrostatic forces to produce nanofibers from a polymer solution containing cupric and ferric ions. By calcining the fibers metal salts are converted to CuFe_2_O_4_ nanoparticles^76^.

Calcination is a post-synthesis thermal treatment that efficiently eliminates volatile substances, moisture, and residual impurities from the synthesized material^77^. In the fabrication of CuFe_2_O_4_ nanoparticles, this treatment is instrumental in controlling final product characteristics, including phase purity, crystallinity, and particle size. To enhance magnetic performance, a common strategy involves elevating the annealing temperature to stabilize the cubic spinel phase^62^. However, at calcination temperatures above approximately 800 °C, the spinel phase may decompose into α-Fe_2_O_3_ and CuO, causing a decrease in *M*_*s*_^47,48,69^.

#### Doping ions into CuFe_2_O_4_ nanostructures

A prominent strategy for engineering is doping, which involves substituting various metal ions into the CuFe_2_O_4_ spinel lattice or substituting Cu^+2^ ions in other ferrite compositions^22,78,79^. Doping CuFe_2_O_4_ with other metal ions can significantly modify the electronic structure and lattice parameters of the crystal. This process induces the creation of new energy states within the band gap, impacts the crystallite size, and modifies the distribution of cations within the spinel framework. These structural and electronic variations play a crucial role in determining magnetic properties, including *M*_*s*_, *H*_*c*_, *M*_*r*_, and magnetic anisotropy, thereby tailoring the material’s performance for targeted applications^80^. Notably, calcination is generally necessary or highly recommended to ensure efficient dopant incorporation and to achieve the desired structural and functional properties of the doped CuFe_2_O_4_ nanoparticles^81^.

Cobalt doping in CuFe_2_O_4_ nanoparticles introduces a smaller, magnetically hard ion that preferentially occupies the B site. This results in stronger A–B superexchange interactions, enhanced *M*_*s*_ and *H*_*c*_, and improved thermal stability, which is considerable for magnetic hyperthermia^47,80,82^. Nickel ions also exhibit a strong preference for occupying the B site in spinel ferrites. Doping CuFe_2_O_4_ nanoparticles with Ni^2+^ significantly enhances several critical characteristics, including magnetic anisotropy, coercivity, electron conductivity, electrode–electrolyte interactions, and catalytic properties^83^. Low-Ni content nanoparticles (e.g., Ni_0.25_Cu_0.75_Fe_2_O_4_) were synthesized to serve as biocompatible catalytic agents^24,84^.

Manganese ions are capable of occupying both A and B sites in the crystal structure; however, they show a preference for the B sites. Mn^2+^ doping results in a notable decrease in the band gap and the crystallite size of CuFe_2_O_4_ nanoparticles, along with an enhancement in their crystallinity. Incorporation of Mn^2+^ ions into the CuFe_2_O_4_ lattice induces the creation of sub-gap energy states, which enhance light absorption, extend the absorption spectrum, and improve photocatalytic performance^81^.

Zn–doping is employed to tune the magnetic softness of CuFe_2_O_4_ nanoparticles. As a diamagnetic ion, Zn^2+^ preferentially substitutes Cu^2+^ ions at A sites, increasing the population of Cu^2+^/Fe^3+^ ions at B sites. This redistribution weakens A–B superexchange interactions, resulting in a substantial decrease in *H*_*c*_ and *M*_*r*_. The net *M*_*s*_ may show a non-monotonic change, often peaking at a specific Zn^2+^ concentration before declining. Concurrently, Zn^2+^ incorporation can inhibit crystal growth during synthesis, yielding nanoparticles with smaller primary size and increased specific surface area^80,85^. This platform demonstrates promising biocompatibility and anticancer activity^86^.

Hu et al.^87^ synthesized CuFe_2_O_4_ nanoparticles doped with titanium and tin ions using a sol–gel/precipitation method^87^. The incorporation of Ti^4+^ and Sn^4+^ ions resulted in a reduced band gap and an increased surface area, which enhanced the catalytic performance of the nanoparticles. Specifically, Ti_0.2_Sn_0.3_Cu_0.5_Fe_2_O_4_ nanoparticles demonstrated the potential to eliminate the HeLa cancer cell line, serving as an effective carrier for the anticancer drug cisplatin. These nanoparticles exhibited a synergistic effect between photothermal and chemotherapy under laser irradiation. Moreover, a high transverse relaxivity (*r*_2_ = 643.1 mM^−1^ s^−1^) indicates that these nanoparticles are excellent candidates for use as T_2_-weighted contrast agents in susceptibility MRI^87^.

#### Engineering CuFe_2_O_4_-based nanostructures

##### Surface engineering for system integration

Surface engineering constitutes a critical design phase for CuFe_2_O_4_-based biomedical platforms, directly addressing key performance barriers including colloidal instability, cytotoxicity, and multifunctionality^88,89^. Unlike uncoated CuFe_2_O_4_ nanoparticles—which are prone to aggregation and uncontrolled Cu^2+^ leaching—engineered surfaces enhance colloidal stability, lower surface energy, and suppress oxidation and aggregation^20,21,88,90–92^. For instance, research demonstrates that a citrate coating on CuFe_2_O_4_ nanoparticles enhances their *M*_*s*_, heating capability, and colloidal stability, which is advantageous for hyperthermia therapy^61^.

Furthermore, surface modifications install reactive chemical groups (such as hydroxyl, carboxyl, amine, or phenol) that enable covalent attachment of targeting ligands, therapeutic agents, or sensor elements, transforming nanoparticles into programmable interfaces for micro/nanosystems^30,31,92–94^. Coating CuFe_2_O_4_ nanoparticles with bovine serum albumin (BSA) strategically meets multiple engineering needs at once. BSA creates a biocompatible protein corona that ensures excellent colloidal stability in physiological conditions, prolongs blood circulation, and shields the surface from immune detection. As a chemical platform, BSA exposes reactive amino and carboxyl groups for covalent linking of targeting ligands or drug molecules^29,95^.

##### Hybrid systems for multifunctional platforms

CuFe_2_O_4_ nanoparticles serve as versatile core templates for constructing multifunctional nanocomposites through heterogeneous nucleation, layer-by-layer deposition, or encapsulation strategies^89^. This architectural flexibility enables integration with plasmonic nanoparticles (Ag), photocatalysts (ZnO), controlled-release matrices (mesoporous silica), or bioactive molecules (drugs, peptides, polymers, fluorescent labels), yielding multifunctional platforms for combined magnetic-therapeutic operation^25,27,32,61,88^. Representative CuFe_2_O_4_-based nanostructures across biomedical applications are summarized in Table 2.

**Table 2:** Synthesis strategies and key properties of engineered CuFe_2_O_4_-based nanoparticles for biomedical applications

Biomedical application	Synthesized nanostructure	Synthesis method	Surface functionalization/coating	Average particle size (nm)	*M*_*s*_ (emu/g)	Ref.
Anticancer (MCF-7 cells)	CuFe_2_O_4_	Sol–gel self-combustion	N/A	56	31	^102^
Anticancer (AGS, MCF-7 cells)	CuFe_2_O_4_@Ag	Green synthesis	*Spirulina platensis*	150	N/A	^32^
Anticancer (HeLa cells)	CuFe_2_O_4_@PSMA-MB	Hydrothermal	Methylene blue-immobilized (MB) Poly(styrene-alt-maleic acid) sodium salt solution (PSMA) shell	21.33 ± 9.24 (particle size) 22.35 ± 13.36 (thickness of shell)	20.75	^25^
Anticancer (A549 tumors in vivo)	CuFe_2_O_4_/GV/LBL (CuFe_2_O_4_@GC-VitE)	Solvothermal	Vitamin E Succinate-glycol (G) Chitosan (C)	200	30.1	^89^
Radiotherapy enhancer (MCF-7 cells)	CuFe_2_O_4_	Coprecipitation/hydrothermal	N/A	30	0.6	^104^
Radiosensitizer & drug delivery (Curcumin)	CuFe_2_O_4_@BSA-FA-CUR	Coprecipitation	Bovine serum albumin (BSA) Folic acid (FA)	70	1	^29^
Drug delivery (Cisplatin)	CuFe_2_O_4_/silica/CDDP	Wet impregnation technique	Monodispersed spherical hydrophilic silica (HYPS)	N/A	7.65	^88^
Drug delivery (Naproxen)	CuFe_2_O_4_@PCL/Col-NPX	Solvothermal	Polycaprolactone (PCL) Collagen (Col)	18.75	80.56	^27^
Hyperthermia & drug delivery	CuFe_2_O_4_/DOX	Green synthesis	*Agaricus bisporus*	110	21.5	^112^
Magnetic hyperthermia therapy	CuFe_2_O_4_@PEG	Hydrothermal Solvothermal	Polyethylene glycol (PEG)	8.4 13.4	24.5 32.7	^58^
Magnetic hyperthermia therapy	Cu_0.4_Zn_0.6+y_Zr_y_Fe_2–2y_O_4_ *y* = 0.05 *y* = 0.1	Citrate sol–gel	N/A	20.8 27.6	31 15	^52^
Magnetic hyperthermia therapy	CuFe_2_O_4_@SiO_2_	Sol–gel combustion	Mesoporous silica	120 (particle size) 14 (thickness of shell)	13.51–7.24	^139^
MRI contrast agent	CuFe_2_O_4_@NH_2_/PVP	Microwave-assisted solvothermal	Monoethanolamine Polyvinylpyrrolidone (PVP)	20	15	^31^
Temperature sensors for MRI	Cu_0.35_Zn_0.65_Fe_2_O_4_	Solid-state sintering	N/A	30	N/A	^117^
MRI thermometry	Cu_0.08_Zn_0.54_Fe_2.38_O_4_@PEG	Thermal decomposition	PEG	20–35	N/A	^26^
Antibacterial (*Escherichia coli, Klebsilla pneumonia, Staphylococcus aureus*, and *Bacillus subtilis*)	Cu_0.5_Ni_0.5_Fe_2_O_4_@PVDF	Combustion	*Aloe barbadensis* Polyvinyldifluoride (PVDF) N-methyl-2-pyrrolidinone (NMP)	54.62	22.85	^73^
Antibacterial (*E. coli and S. aureus*)	CuFe_2_O_4_	Coprecipitation	N/A	50 − 100	21.39	^122^
Antibacterial (*S. aureus*, *E. coli* and *C. albicans*)	CuFe_2_O_4_@Cit CuFe_2_O_4_/ZnO@Cit	Microwave-activated solvothermal	Citrate functionalized	10	52 29	^61^
Antibacterial (*B. subtilis* and *P. aeruginosa*)	C@CuFe_2_O_4_	Solvothermal	In-situ generated CO_2_	30–40	N/A	^120^
Antibacterial (*B. subtilis, S. aureus, E. coli*, and *Salmonella choleraesuis*)	CuFe_2_O_4_/zeolite	Coprecipitation	Zeolite framework	50	10−41	^125^
Antibacterial (*S. aureus*)	CuFe_2_O_4_@CA-LEO	Solvent/anti-solvent technique	Cellulose acetate Lemongrass essential oil	70 (particle size) 220 (capsule size)	N/A	^119^
Antibacterial (*Candida albican* and *Pseudomonas aeruginosa*)	CuFe_1.95_Y_0.05_O_4_ CuFe_1.95_Sm_0.05_O_4_	Coprecipitation	N/A	10–60 nm	N/A	^140^
Nutritive supplement for cucumber plants	CuFe_2_O_4_	Coprecipitation	N/A	N/A	25.5	^60^
Wound healing	CuFe_2_O_4_@chitosan/PEO	Coprecipitation	Poly(ethyleneoxide) (PEO) Chitosan	N/A	N/A	^30^
Scaffold for bone tissue	CuFe_2_O_4_/Mg_2_SiO_4_@P3HB	Sol–gel combustion	Poly-3-hydroxybutyrate (P3HB)	100–200	N/A	^127^
Vascular graft (Antioxidant)	CuFe_2_O_4_@PU	Microwave- assisted hydrothermal	Polyurethane (PU)	5	N/A	^19^

Reported synthesis methods arise from disparate experimental conditions and material systems and are not directly comparable. Similarly, saturation magnetization (*M*_*s*_) values are highly dependent on measurement conditions, including field strength, temperature, and sample composition. These variations should be considered when comparing data across studies

A widely explored configuration for engineered nanocomposites is the core–shell architecture, which tunes relaxation dynamics for optimized transduction in lab-on-chip platforms^96^. Garfami et al.^32^ investigated the anticancer activity of CuFe_2_O_4_@Ag core-shell nanohybrids against gastric adenocarcinoma (AGS) and breast cancer (MCF-7) cell lines^32^. The cubic phase CuFe_2_O_4_ core was synthesized via coprecipitation, after which a silver shell was deposited using a green approach with *Spirulina platensis*. The organic components from the extract, including amine and phenol groups, facilitated shell formation and provided colloidal stability, as confirmed by FTIR and zeta potential analysis (-30.6 mV). The engineered nanocomposites exhibited potent and dose-dependent cytotoxicity, with half-maximal inhibitory concentration (IC_50_) values quantified at 180 µg/mL for MCF-7 and 220 µg/mL for AGS cells. The apoptotic mechanisms of these nanocomposites proceeded without eliciting a necrotic or inflammatory response in surrounding tissue, underscoring the selectivity of the engineered nanotherapeutic system^32^.

#### Characterization of CuFe_2_O_4_ nanostructures

To develop reliable CuFe_2_O_4_ nanoparticles, a comprehensive characterization strategy is crucial for establishing clear correlations between structure, properties, and performance. This integrated approach allows precise regulation of particle size (analyzed by electron microscopy), surface modification and colloidal stability (evaluated through dynamic light scattering and zeta potential measurements), crystal structure and purity (determined by X-ray diffraction), and drug-loading efficiency (examined by thermogravimetric analysis). The resulting analytical insights support the rational design of CuFe_2_O_4_-based nanostructures with predictable functionality across diverse biomedical applications (Table 3)^97–99^.

**Table 3:** Characterization techniques for CuFe_2_O_4_ nanostructures: Measured parameters, material-specific metrics, and functional relevance

Category	Technique	Key parameters measured	CuFe_2_O_4_-specific metrics	Engineering relevance	Ref.
Morphology	Field emission scanning electron microscopy (FE-SEM)/transmission electron microscopy (TEM)	Particle size, shape, and Shell thickness	~10–140 nm; spherical, cubic, or irregular	Coating thickness vs magnetic volume trade-off	^59,68,104,132,141,142^
Colloidal	Dynamic light scattering (DLS)/ζ-potential	Hydrodynamic diameter, PDI, surface charge stability	~50–300 nm; >±30 mV for stable nanoparticles	Predicts circulation time, aggregation risk Confirms coating success, serum stability	^18,26,112^
Structure	X-ray diffraction (XRD)	Crystal phase, crystallite size, lattice parameter	Cubic/tetragonal; ~7–50 nm; ~8.36–8.39 Å (cubic)	Anisotropy vs superparamagnetism tuning	^20,40,44,70,77,112,137^
Chemistry	Fourier transform infrared spectroscopy (FTIR)	Metal-oxygen bonds, dopant ions, and functional groups	Fe–O ~ 400–600 cm^-1^; Cu–O ~ 400–500 cm^−1^	Confirms spinel integrity, surface groups	^61,143–146^
	X-ray photoelectron spectroscopy (XPS)	Surface elemental composition, oxidation states	Cu^2+^/Cu^+^ ratio; ligand bonding	Quantifies conjugation efficiency	^98^
Magnetic	Vibrating sample magnetometry (VSM)	*M*_*s*_, *M*_*r*_, *H*_*c*_, and blocking temperature	*M*_*s*_: ~20–80 emu/g; *H*_*c*_: ~50–300 Oe	Explores magnetic properties of nanoparticles and hyperthermia field compatibility	^27,59,60,147–149^
Thermal	Thermogravimetric analysis (TGA)/differential scanning calorimetry (DSC)	Organic loading, phase transitions	Crystallization (~250–350 °C); phase transitions (~360–450 °C); decomposition (above 800 °C)	Optimize loading percentage of the organic shell or therapeutic cargo	^46,150,151^
Optical	UV-Vis	Drug loading, plasmonic effects	Fe/Cu ratio effects	Verifying the successful synthesis Release kinetics, loading quantification	^25^

#### Biomedical applications of CuFe_2_O_4_ nanostructures

CuFe_2_O_4_ nanostructures are engineered as multifunctional theranostic platforms^100^. Owing to their intrinsic physicochemical properties, they facilitate precise applications such as high-efficiency MHT, field-responsive drug release, enzyme-mimetic biosensing, MRI contrast enhancement, and ROS-mediated antimicrobial or anticancer activity (Table 4)^21,26^. This integrated functionality transforms passive nanomaterials into responsive agents. Notably, at low concentrations, they exhibit selective cytotoxicity toward diseased cells while preserving overall biocompatibility, thereby ensuring an optimal balance between therapeutic efficacy and safety^27,32^.

**Table 4:** Biomedical applications of CuFe_2_O_4_ nanoparticles and their mechanisms of action

Biomedical applications	Mechanisms	Dosage of CuFe_2_O_4_	Ref.
Cancer therapy	○ Cell membrane interaction ○ Fenton/Fenton-like reactions ○ *BAX* upregulation ○ Mitochondrial depolarization ○ ROS generation ○ GSH depletion ○ CAT decreasing ○ Biomolecule damage ○ DNA fragmentation ○ Chromatin condensation ○ Nuclear disintegration ○ Lipid peroxidation ○ Apoptosis induction ○ Radiotherapy enhancement ○ PTT synergy ○ MHT synergy	10–250 μg/mL	^25,29,32,76,95,102–104^
Magnetic hyperthermia treatment (MHT)	○ Localized heat production ○ Protein denaturation ○ Enhanced drug uptake ○ Apoptosis triggering ○ Synergy with photothermal/chemotherapy	1–10 mg/mL	^58,61,79,92,109,111^
Targeted drug delivery	○ Chemodynamic therapy synergy ○ PH-responsive release ○ Surface functionalization	0.5–10 mg/mL	^27,88,112^
Biosensing	○ Nanozyme-like catalysis ○ High sensitivity ○ Target-specific signal generation	1–5 mg/mL	^91^
Magnetic resonance imaging (MRI)	○ Local magnetic field inhomogeneities ○ Surface modification for targeting ○ Reduced toxicity ○ Enhanced safety	20–200 μg/mL	^26,31,117^
Antibacterial effects	○ Membrane disruption ○ Cytoplasmic leakage ○ Fenton/Fenton-like reactions ○ ROS generation ○ Biomolecule damage ○ Bactericidal action ○ Anti-biofilm ○ Proliferation inhibition ○ MHT synergy	100– 1000 μg/mL	^22,33,61,119,120,122–125^

##### Anticancer

**Fig. 5:**
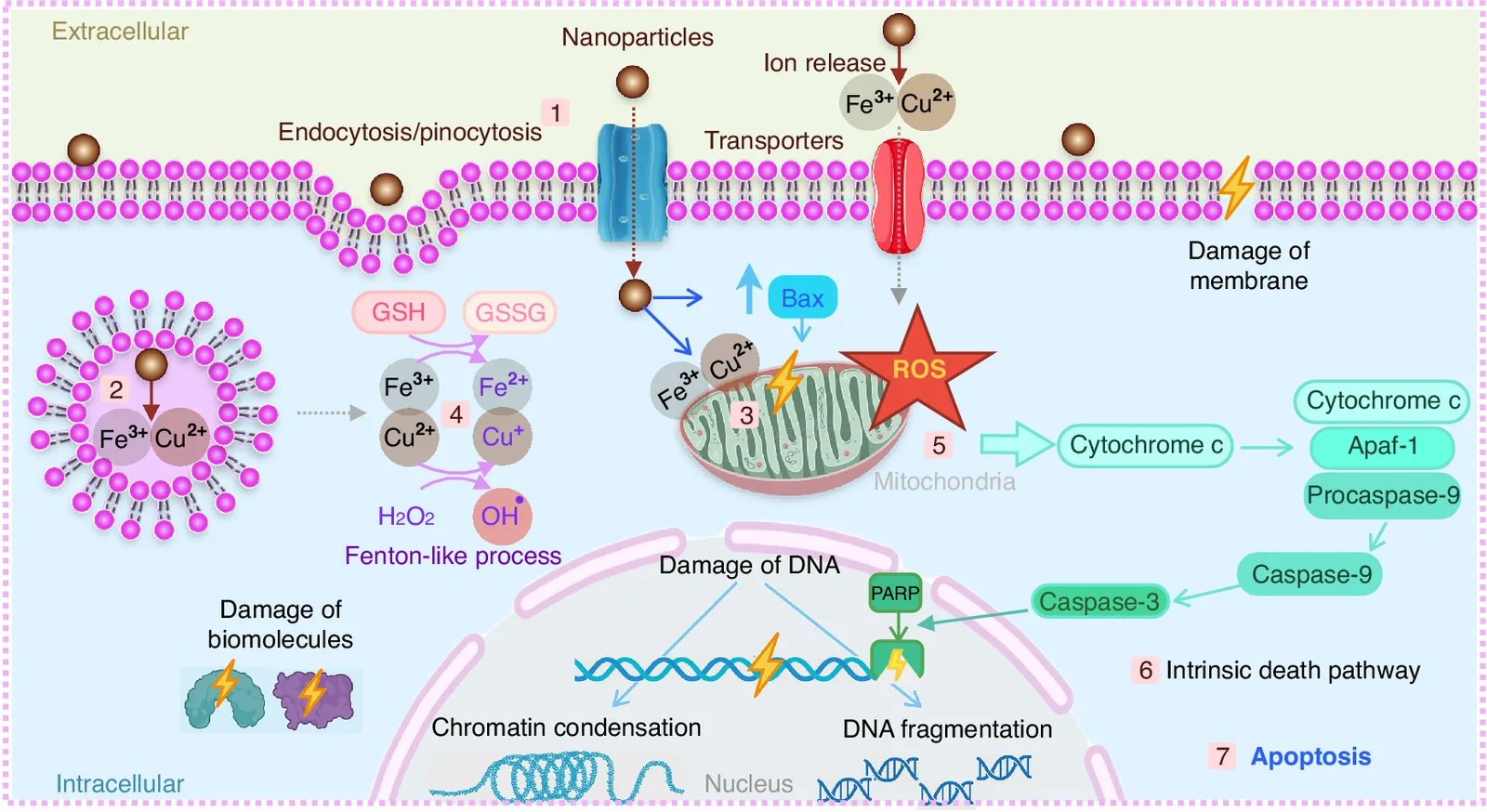
**Schematic of CuFe2O4-mediated chemodynamic therapy (CDT)**. (1) Cellular internalization of nanoparticles (via endocytosis/transporters) and ions (via transporters); (2) intracellular release and accumulation of Cu^2+^/Fe^3+^ ions (3) disruption of mitochondrial membrane potential and function; (4) Fenton/Fenton-like process, GSH depletion, (5) generation of ROS, and release of cytochrome c; culminating in (6) activation of the intrinsic (mitochondrial) apoptosis pathway, leading to (7) apoptosis

CuFe_2_O_4_ nanoparticles are engineered to exploit distinct cancer cell vulnerabilities through multimodal therapeutic actions^101^. These nanoparticles can selectively exert cytotoxic effects attributed to their ability to interact with cancer cell membranes^32^. Their foundational anticancer mechanism is chemodynamic therapy (CDT) via synergistic bimetallic Fenton/Fenton-like reactions that trigger ferroptosis and cuproptosis pathways^95^. The coexistence of copper and iron engages in a reciprocal redox cycle. In this cycle, the Cu^+^/Cu^2+^ redox pair efficiently regenerates the active Fe^2+^ catalyst from Fe^3+^, circumventing the slow reduction step that constrains ROS yield in iron-only systems. This catalytic partnership generates a potent, continuous stream of hydroxyl radicals (•OH) within the mildly acidic (pH ~ 6.5) tumor microenvironment, where Cu^+^ activity remains high. Simultaneously, CuFe_2_O_4_ nanoparticles induced a significant increase in lipid peroxidation (LPO), glutathione S-transferase (GST), and glutathione peroxidase (GPX) levels, while decreasing catalase (CAT) activity and glutathione (GSH) content. This ROS burst induces mitochondrial dysfunction, biomolecular oxidation, nuclear fragmentation, and apoptosis in malignant cells (Fig. 5)^25,76,95,101–103^.

Hu et al.^89^ engineered a tumor-specific CuFe_2_O_4_-based nanocomposite to selectively induce cytotoxic effects in cancer cells^89^ The architecture was constructed through a multi-layer assembly. First, redox-active CuFe_2_O_4_ magnetic cores were synthesized via a solvothermal method. These were then coated with a vitamin E succinate–glycol chitosan (GV) copolymer, engineered to be enzymatically cleaved by esterases. Subsequently, a polylysine interlayer and a human serum albumin outer shell were added to confer pH-responsive structural disassembly in acidic tumor microenvironments and active tumor targeting via overexpressed albumin receptors, particularly in lung cancer. The GV polymer coating served as a smart metabolic switch, enabling differential oxidative regulation in tumor versus normal cells. In normal cells with high esterase activity, the polymer was efficiently cleaved, releasing vitamin E derivatives that acted as potent antioxidants to buffer ROS and protect cellular redox balance. This effectively shielded normal tissues from oxidative damage. Conversely, in cancer cells—which typically exhibit lower esterase activity and elevated baseline ROS—the polymer remained largely intact, thereby augmenting the intrinsic oxidative stress generated by the CuFe_2_O_4_ core. Cellular assays confirmed this selectivity, showing significantly lower IC_50_ values in lung cancer cell lines compared to normal epithelial or kidney cells. In xenograft mouse models of lung cancer, repeated intravenous administration of the nanocomposites led to pronounced, dose-dependent tumor growth suppression. Critically, despite accumulation in the liver and spleen (typical of nanoparticles cleared by the reticuloendothelial system), no significant organ toxicity or hematological abnormalities were observed, demonstrating an acceptable safety profile. Biodegradability was primarily mediated through enzymatic cleavage of the chitosan-based polymer and gradual dissolution of metal ions from the ferrite core. The organic components were metabolized into biocompatible byproducts, whereas the released trace metals are processed through physiological pathways. The combination of degradable coatings and controlled ion release reduces long-term accumulation risks, addressing a common limitation of inorganic nanomaterials^89^.

A secondary anticancer mechanism of CuFe_2_O_4_ exploits the radiosensitizing properties of its superparamagnetic nanoparticle form. Under X-ray irradiation, these nanoparticles enhance localized energy deposition within tumor tissue via effects such as the photoelectric effect and Auger electron emission. This targeted action amplifies CDT efficacy while preserving a favorable cytotoxicity profile for healthy cells^29,104^.

The work of Salehiabar et al.^29^ exemplifies the systematic engineering of CuFe_2_O_4_ nanoparticles into a multifunctional theranostic platform for synergistic cancer therapy^29^. The researchers employed a rational, layer-by-layer design strategy: a CuFe_2_O_4_ magnetic core was functionalized with a BSA coating to impart colloidal stability and biocompatibility, conjugated with folic acid (FA) for active tumor targeting, and loaded with curcumin (CUR) as a chemotherapeutic and radiosensitizing agent. In vitro evaluation using 4T1 mouse breast cancer cells confirmed the platform’s favorable biosafety profile, intrinsic cytotoxicity, and, most notably, its potent capacity to amplify the effects of radiotherapy. The CUR release kinetics exhibited a sustained/controlled way, accelerating drug elution under the acidic conditions typical of the tumor microenvironment compared to physiological pH. Furthermore, the nanosystem demonstrated exceptional hemocompatibility, with hemolysis rates remaining below 4% even at high concentrations. The most compelling validation came from in vivo studies in a 4T1-tumor-bearing BALB/c mouse model. The synergistic combination of the CuFe_2_O_4_@BSA-FA-CUR nanoplatform with a single, moderate dose of X-ray irradiation (4 Gy) resulted in complete tumor ablation in nearly all treated subjects. Crucially, this remarkable therapeutic efficacy was achieved without inducing systemic toxicity or observed radiation damage to healthy organs, highlighting the success of its targeted design and biocompatible engineering^29^. This study provides a powerful paradigm for transforming CuFe_2_O_4_ from a simple nanomaterial into a sophisticated, targeted system for combinatorial therapy, effectively bridging material properties with transformative clinical potential.

Furthermore, CuFe_2_O_4_ nanoparticles can be engineered as photothermal therapy (PTT) conversion agents, absorbing near-infrared (NIR) light to generate localized tumoricidal/bactericidal heat^105^. PTT is widely regarded as a highly promising form of hyperthermia owing to its high level of externally controlled specificity and exceptional safety^106^. One example is a phototherapeutic system where methylene blue, an FDA-approved photosensitizer, is immobilized onto CuFe_2_O_4_ nanoparticles via a hydrothermal synthesis route to form a functional polymer shell^25^. Under 660 nm light irradiation, the composite efficiently generates localized ROS, inducing potent cytotoxic effects against cancer cells and improving therapeutic outcomes. Furthermore, the acidic tumor microenvironment promotes a controlled dissolution of Cu^+2^ and Fe^3+^ ions from the ferrite core. This ion release provides a direct biodegradation pathway, mitigating the risk of long-term nanoparticle accumulation^25^.

##### Magnetic hyperthermia therapy (MHT)

**Fig. 6:**
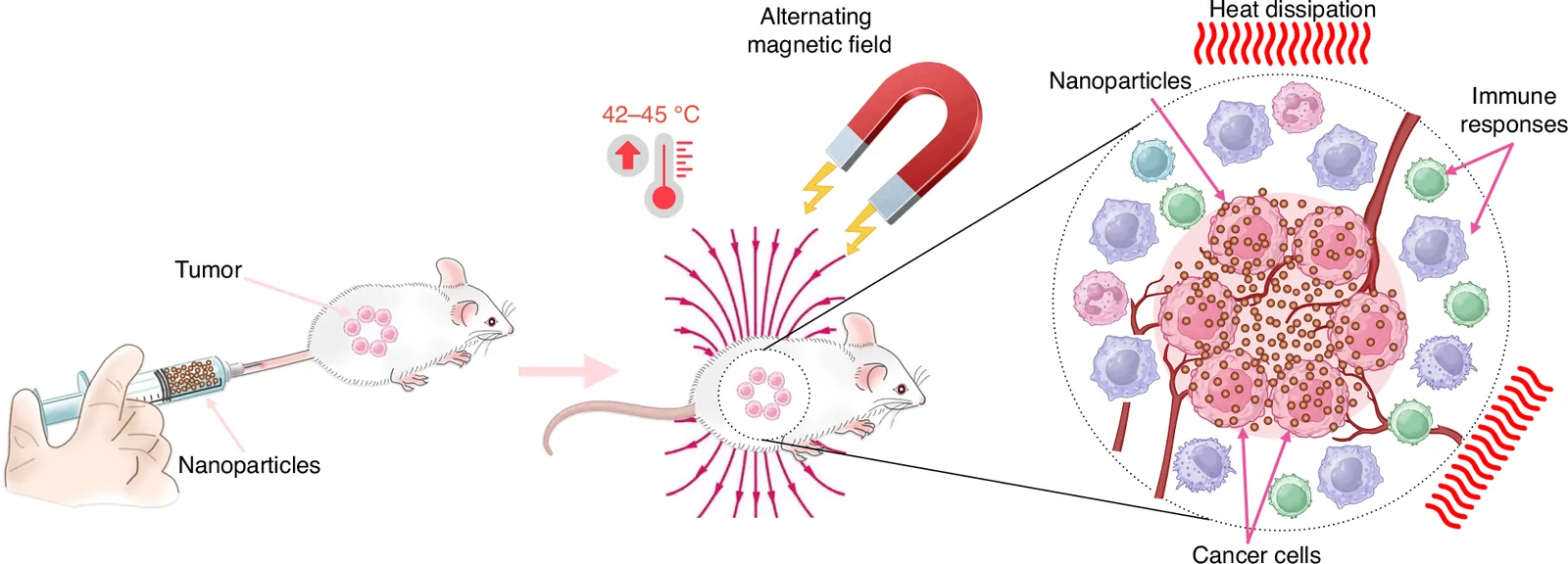
**Schematic of MHT mediated by CuFe2O4 nanoparticles**. Following intravenous administration, nanoparticles are magnetically guided to the tumor. Subsequent application of an AMF induces localized heating via magneto-thermal energy conversion

Recent research highlights CuFe_2_O_4_ nanoparticles as a promising platform for MHT, offering a favorable combination of heating efficiency, tunable magnetism, and low anisotropy. In MHT, an externally applied alternating magnetic field (AMF) activates the nanoparticles to generate localized heat via magneto-thermal energy conversion, inducing protein denaturation and triggering apoptosis in target cells (cancer or bacterial cells)^61,107^. The therapeutic efficacy is achieved within a defined thermal window (42–45 °C), which exploits the heightened thermal sensitivity of malignant cells (Fig. 6)^108^. The hyperthermic function can be further integrated with other modalities, such as photothermal or chemotherapy, to produce synergistic apoptotic effects and enhance drug permeability^109^.

The specific absorption rate (SAR) quantifies heating performance, defined as the thermal power dissipated per unit mass of magnetic material The heating efficiency for magnetic hyperthermia is quantified by the specific absorption rate (SAR, W/g), which measures the thermal power dissipated per unit mass of magnetic material (SAR = C·V_s_/m · dT/dt, Where *C* is the sample’s specific heat capacity, *V*_*s*_ is the sample volume, *m* is the mass of magnetic material, and *dT/dt* is the initial rate of temperature rise)^110^.

The *M*_*s*_ of magnetic nanoparticles is a crucial parameter influencing their thermal energy dissipation when subjected to AFM^58^. While hard ferrites exhibit high *M*_*s*_, they also possess high coercivity, requiring stronger AFM to overcome magnetic hardness and achieve sufficient SAR for effective hyperthermia. This presents a clinical challenge due to patient safety constraints on applied field strength and frequency. CuFe_2_O_4_ nanostructures outperform harder ferrites in MHT due to moderate *M*_*s*_, low coercivity, and biocompatibility, enabling efficient heating within H·f safety limit (<5 × 10^9 ^A/m·s)^24,58,111^.

Jamir et al.^92^ improved heating efficiency by leveraging exchange coupling between magnetically hard and soft phases, optimizing magnetic anisotropy for enhanced energy conversion within safe limits^92^. In their study, cubic Cu_x_Co_1-x_Fe_2_O_4_ nanoparticles (where *x* = 0.15, 0.31 and 0.47) were synthesized via solvothermal and coated with chitosan. Cu^2+^ doping reduced both *H*_*c*_ and *M*_*r*_, while *M*_*s*_ initially increased, peaking at *x* = 0.31. The optimized Cu_0.31_Co_0.69_Fe_2_O_4_ nanoparticles demonstrated exceptional hyperthermic performance. At a concentration of 1 mg/mL and under a moderate applied field (337 kHz, 187 Oe), the nanoparticles achieved a high SAR of 446 W/g and induced a temperature rise to 48 °C^92^.

The strategic incorporation of Cu^2+^ ions into softer ferrite matrices represents a powerful method for fine-tuning magnetic properties to enhance hyperthermia performance under clinically safe field conditions. Ansari et al. demonstrated this by systematically doping ZnMg-ferrite (Cu_x_Zn_0.5–x_Mg_0.5_Fe_2_O_4_ where *x* = 0–0.5) via hydrothermal synthesis with polyvinyl alcohol (PVA) capping^79^. Characterization showed Cu^2+^ incorporation reduced the lattice constant and increased particle size, while *M*_*s*_ peaked at an optimal *x* = 0.3 composition. This resulting Cu_0.3_Zn_0.2_Mg_0.5_Fe_2_O_4_ composition demonstrated a high heating efficiency, elevating the temperature to 43 °C in 510 s under a mild AMF (200 kHz, 125 Oe). Biocompatibility assays with MG63 cells further indicated that Cu^2+^ content critically modulates cellular compatibility, highlighting stoichiometry as a key design parameter for safe therapeutic agents^79^. This strategy highlights how compositional engineering can balance high heating efficiency with the low-field requirements of safe clinical systems.

##### Targeted drug delivery

CuFe_2_O_4_ nanoparticles constitute a versatile engineering platform for advanced drug delivery systems, integrating magnetic guidance with stimuli-responsive release for enhanced therapeutic precision alongside with synergistic therapeutic effects with the drug^112^. The inherent superparamagnetism of CuFe_2_O_4_ enables external magnetic guidance to influence nanoparticle accumulation at the target site, enhancing spatial precision for therapy. The material’s large surface area and adaptable surface chemistry support loading of a range of therapeutic cargos through hydrogen bonding, hydrophobic interactions, and other multivalent bindings. Moreover, the redox-active surface increases drug loading efficiency, enabling higher payloads compared to non-redox-active ferrites^88^.

Incorporating CuFe_2_O_4_ nanoparticles in a drug delivery system reduces the rate of drug release, maintaining drug concentration in the blood or target tissue at desired values as long as possible, so as to exert control over duration and drug release rate^27^. CuFe_2_O_4_ nanoparticles facilitate pH-triggered drug release via selective dissolution of Cu^2+^ ions in acidic microenvironments, such as the tumor interstitium or endosomal compartments, enabling on-demand payload delivery. This mechanism supports prolonged drug exposure in the target tissue, helping maintain therapeutic concentrations over extended periods^95,113^.

A study by Armedya et al.^27^, exemplifies the rational design of such a stimulus-responsive system^27^. CuFe_2_O_4_ nanoparticles (*M*_*s*_ of 80.56 emu/g) were synthesized through a solvothermal method using benzyl ether and oleylamine as solvent and capping ligand, respectively. These hydrocarbon-functionalized nanoparticles were incorporated into polycaprolactone (PCL)/collagen nanofibers using the electrospinning technique for naproxen delivery. A key feature of this design was the exploitation of distinct material affinities. The hydrophobic, oleylamine-capped nanoparticles were selectively incorporated into the PCL domains of the composite, while the semi-polar naproxen drug molecule interacted with both polymer phases through non-covalent bonds. This design resulted in a tunable release profile, where increased nanoparticle loading enhanced the matrix’s hydrophobicity and directly reduced the diffusion rate of the hydrophilic drug, fine-tuning the release according to the Korsmeyer-Peppas model. Furthermore, the system exhibited pH-responsive behavior, with accelerated release in alkaline conditions due to electrostatic repulsion-induced pore opening within the nanofiber matrix^27^.

A comparative study by Mohana and Sumathi (2024) provides a systematic engineering evaluation of green-synthesized spinel ferrites (ZnFe_2_O_4_, CoFe_2_O_4_, and CuFe_2_O_4_ nanoparticles) for integrated cancer therapy^112^. These nanoparticles were synthesized via a green biosynthetic method using *Agaricus bisporus* (white button mushroom) extract. In cytotoxicity assessments against SW620 colon cancer cells, CuFe_2_O_4_ exhibited the most potent effect, reducing cell viability to 31% versus 42.5% and 49.3% for ZnFe_2_O_4_ and CoFe_2_O_4_, respectively. This enhanced cytotoxicity correlated with superior intrinsic properties, including the highest recorded antioxidant activity among the three ferrites. In MHT, CuFe_2_O_4_ also demonstrated the most efficient heating profile. At a concentration of 10 mg under a field of 235 A/m and 335 kHz, it achieved the therapeutic threshold of 44 °C in just 31 s, significantly faster than CoFe_2_O_4_ (122 s). This performance yielded a high SAR of 337 W/g for CuFe_2_O_4_. For drug delivery, doxorubicin (DOX) was loaded onto the nanoparticles primarily via hydrogen bonding and hydrophobic interactions. Release kinetics were tunable by nanoparticle concentration and composition: 10 mg of CuFe_2_O_4_, ZnFe_2_O_4_, and CoFe_2_O_4_ released 99.52%, 98.74%, and 97.34% of their DOX payload within 6 h, respectively. Reducing the nanoparticle concentration decreased the release rate, a design feature that can minimize premature drug loss in circulation and mitigate off-target toxicity. Collectively, these data position green-synthesized CuFe_2_O_4_ as a high-performance multi-modal platform, outperforming its zinc and cobalt analogs in key therapeutic metrics for combinatorial hyperthermia and chemotherapy^112^.

##### Biosensing

The properties of CuFe_2_O_4_ nanoparticles collectively enable efficient enzyme immobilization and signal amplification, positioning these nanoparticles as a high-performance transducer material. Surface functionalization using silane-based coupling agents facilitates stable enzyme attachment while preserving catalytic activity, thereby improving interfacial charge transfer and analytical sensitivity. Notably, the nanozyme-like behavior of CuFe_2_O_4_ contributes directly to signal enhancement by catalyzing electrochemical reactions associated with target analytes, a feature repeatedly correlated with improved detection^83,91,114^.

A representative example is provided by the work of Sanko et al.^91^, who developed an electrochemical biosensor for urea detection in complex matrices such as soil and milk^91^. In their investigation, several spinel ferrites, including CoFe_2_O_4_, NiFe_2_O_4_, ZnFe_2_O_4_, and CuFe_2_O_4_, were synthesized via the coprecipitation method and subsequently amine-functionalized with tetraethoxysilane (TEOS) and 3-aminopropyl-triethoxysilane (APTES). These modified nanoparticles were integrated into a polyaniline/Nafion composite deposited on a glassy carbon electrode, followed by immobilization of urease as the biorecognition element. The sensing mechanism relies on enzymatic hydrolysis of urea into ammonium (NH_4_^+^) and bicarbonate ions. While these hydrolysis products are intrinsically electroinactive, CuFe_2_O_4_ nanoparticles act as electrocatalysts that facilitate the oxidation of NH_4_^+^ ions, thereby generating a measurable current response. This catalytic mediation represents the core transduction mechanism and obviates the need for additional redox enzymes. Electrochemical characterization using cyclic voltammetry and differential pulse voltammetry revealed a pronounced increase in oxidation peak currents for CuFe_2_O_4_-based electrodes—approximately six- to seven-fold higher than non-ferrite controls—along with notable shifts in oxidation potential. Among all tested ferrites, CuFe_2_O_4_ exhibited the highest sensitivity and signal amplification. The superior performance of CuFe_2_O_4_ was attributed to its comparatively narrow bandgap, which promotes efficient electron transfer between enzymatic reaction products and the electrode surface. This electronic advantage enhances charge carrier mobility and lowers the activation energy for electrooxidation processes, resulting in heightened biosensor responsiveness. Accuracy was validated through spike-and-recovery experiments at micromolar concentrations, demonstrating reliable analytical performance across replicate measurements^91^.

##### Magnetic resonance imaging (MRI)

Magnetic resonance imaging (MRI) contrast arises from differences in proton relaxation behavior, primarily governed by longitudinal (T_1_) and transverse (T_2_) relaxation times. T_1_ relaxation reflects the recovery of longitudinal magnetization following radiofrequency excitation, whereas T_2_ relaxation describes the irreversible decay of transverse magnetization due to spin–spin interactions. In practical imaging systems, an additional parameter—T_2_*—is often measured, which incorporates both intrinsic T_2_ decay and extrinsic dephasing effects caused by local magnetic field inhomogeneities. As a result, T_2_* is always shorter than T_2_ and is particularly sensitive to magnetic susceptibility effects introduced by iron-based nanoparticles^115^.

Most ferrite nanoparticles, including CuFe_2_O_4_, predominantly act as negative contrast agents, accelerating transverse relaxation through the generation of nanoscale magnetic field gradients. Consequently, their MRI performance is more accurately characterized by r_2_ and r_2_* relaxivities rather than r_1_ alone. Importantly, studies reporting T_2_*-weighted imaging should not be interpreted as equivalent to spin–spin T_2_ relaxation, as gradient-echo–based T_2_* contrast emphasizes susceptibility-driven dephasing rather than intrinsic spin interactions. A clear distinction between these mechanisms is therefore essential when evaluating and comparing MRI performance across studies^31,116^.

CuFe_2_O_4_ nanoparticles exhibit several features that distinguish them from conventional ferrite-based MRI agents. The presence of Jahn–Teller distortion, which modifies crystal symmetry and promotes spin canting at the nanoparticle surface. This structural asymmetry enhances local magnetic susceptibility variations, thereby strengthening transverse dephasing effects that contribute to T_2_ and T_2_* contrast. Compared with magnetically harder ferrites CuFe_2_O_4_ displays moderate *M*_*s*_ and negligible coercivity at the nanoscale, enabling efficient susceptibility contrast while maintaining compatibility with clinically acceptable magnetic field conditions^31^. In addition, improved colloidal stability of CuFe_2_O_4_ nanoparticles reduces sedimentation-induced signal heterogeneity and enhances signal-to-noise ratio during imaging. These features are particularly advantageous for quantitative MRI, where uniform dispersion and reproducible relaxivity behavior are required for reliable contrast interpretation^26^.

The MRI performance of CuFe_2_O_4_ nanoparticles is highly tunable through nanoscale engineering. Particle size, crystallinity, cation distribution, and surface chemistry directly influence magnetic moment fluctuations and water–proton accessibility, which collectively determine r_2_/r_1_ and r_2_*/r_1_ ratios. Surface functionalization with small molecules (e.g., citrate, glutathione, or ascorbate) or polymers can regulate hydration layers and reduce aggregation, thereby modulating transverse relaxivity and enhancing biocompatibility. Ultrasmall (<10 nm) superparamagnetic CuFe_2_O_4_ cores offer the possibility of dual-mode or switchable contrast, where T_1_-weighted enhancement may dominate at low local concentrations (e.g., in the bloodstream), while T_2_ or T_2_* effects become prominent upon accumulation in target tissues such as tumors or reticuloendothelial system (RES) organs. This spatially and temporally evolving contrast behavior represents a functional advantage over conventional single-mode agents and aligns with the development of integrated, image-guided theranostic nanosystems^31,117^.

Heydari et al.^31^ provide a representative example of how synthesis strategy and surface engineering influence the MRI performance of CuFe_2_O_4_-based nanostructures^31^. In their study, CuFe_2_O_4_ nanostructures were synthesized via a microwave-assisted solvothermal route, employing 350 W for 4 min as a rapid and energy-efficient alternative to conventional solvothermal processing. This method yielded ultrasmall nanoparticles with crystallite sizes of 8 ± 2 nm and superparamagnetic behavior (*M*_*s*_ = 15 emu/g, *M*_*r*_ = 0 emu/g, *H*_*c*_ = 0 Oe). The engineering approach encompassed amine-functionalizing CuFe_2_O_4_ cores attributable to the adsorbed ethylene glycol and monoethanolamine molecules, followed by embedding in polyvinylpyrrolidone (PVP) to enhance steric stabilization and colloidal robustness. MRI measurements were conducted at 3 T using both in vitro and in vivo configurations. The reported r_2_/r_1_ ratio (~0.044) indicates a dominant transverse relaxation effect, consistent with T_2_* contrast behavior in superparamagnetic nanoparticles. In vivo experiments in BALB/c mice involved tail-vein administration of PVP-stabilized CuFe_2_O_4_ nanoparticles at a dose of approximately 9.05 mg/kg body weight. T_2_*-weighted imaging (TR = 350 ms, TE = 1.75–60 ms) revealed rapid hypointense signal changes in the liver and spleen within 10 min post-injection, confirming efficient RES uptake and susceptibility contrast generation. These findings demonstrate that CuFe_2_O_4_ nanoparticles can achieve clinically relevant contrast at standard preclinical doses, while exhibiting biodistribution patterns typical of superparamagnetic iron oxide nanoparticles, providing numerous advantages such as higher signal-to-noise ratios and enhanced spectral resolution, establishing better spatial and temporal resolutions than previously attained^31^.

##### Antimicrobial activity

**Fig. 7:**
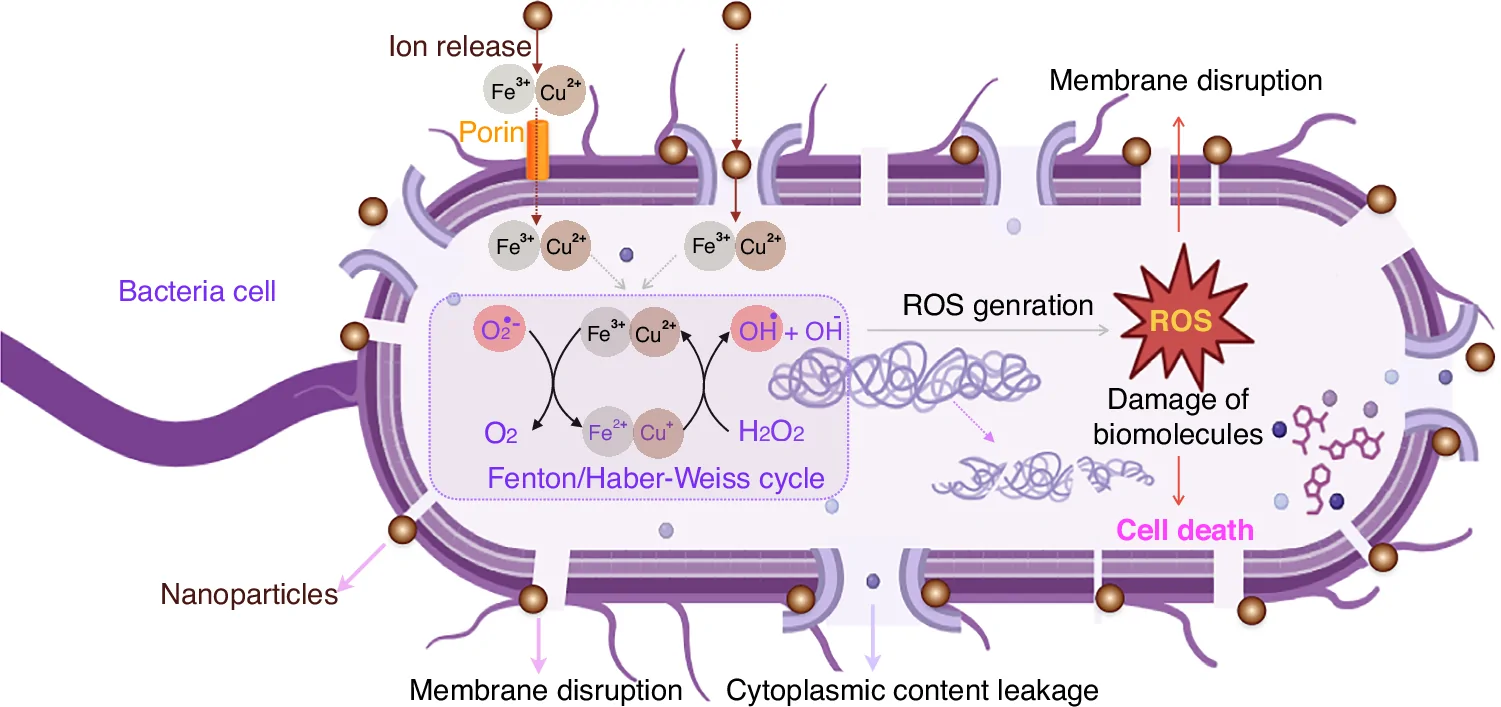
**Proposed antibacterial mechanism of CuFe2O4 nanoparticles**. The nanoparticles attach to and disrupt the bacterial membrane, facilitating the release of metal ions. These ions (Cu^2+^/Fe^3+^) enter the cell via porins and damaged membrane sites. Intracellular ions catalyze Fenton/Haber-Weiss cycles, generating a surge of ROS. The resulting oxidative stress causes extensive biomolecular damage, culminating in membrane failure, leakage of cytoplasmic contents, and cell death

CuFe_2_O_4_ nanoparticles represent an alternative to traditional antibiotics, which face diminishing efficacy as a result of rising antibiotic resistance among bacterial strains^118^. These nanoparticles have garnered significant interest because of their potent bactericidal, antibiofilm, and antiproliferative activities^22,119^. The primary antibacterial mechanism of CuFe_2_O_4_ nanoparticles is the generation of ROS via Fenton and Fenton-like reactions, which damage vital cellular components and further contribute to bacterial cell death^120,121^. A critical secondary mechanism is direct membrane disruption; their nanoscale dimensions and surface properties enable them to attach to and compromise bacterial cell walls and membranes. This interaction induces a contact-killing effect, causing cytoplasmic content leakage and structural collapse (Fig. 7)^33,122,123^. Furthermore, the inherent antibacterial activity of CuFe_2_O_4_ nanoparticles can be amplified through synergistic strategies such as material engineering, PTT, and MHT^33,124^. These combined actions confer broad-spectrum efficacy against both Gram-positive and Gram-negative pathogens.

Liu et al.^100^ developed a multimodal antibacterial platform that integrates sequential physical, chemical, and thermal therapeutic actions^100^. The system is based on hemoglobin-functionalized CuFe_2_O_4_ nanoparticles (*M*_*s*_ = 43.8 emu/g), synthesized via a hydrothermal method, which serve as both Fenton-like nanocatalysts and NIR photothermal agents. These nanostructures effectively catalyze the production of ·OH, inducing oxidative damage that increases bacterial membrane permeability and thermal sensitivity. Upon exposure to 808 nm NIR laser irradiation, these nanoparticles generate localized hyperthermia, leading to the rapid death of pre-damaged bacteria. A key innovation of this platform is the use of magnetic enrichment, which amplified the photothermal effect by approximately 20-fold. At a low particle concentration (20 µg/mL), a temperature rise sufficient for bacterial ablation (>50 °C) was achieved only under magnetic accumulation, thereby enhancing therapeutic efficiency while minimizing off-target effects. In vitro antibacterial assays demonstrated broad-spectrum activity, with 100% eradication of *E. coli* and 96.4% eradication of *S. aureus* under combined catalytic and photothermal treatment. In a murine subcutaneous abscess model infected with *S. aureus*, the synergistic therapy resulted in complete wound healing within 10 days, with scars resolving entirely. Histopathological analysis confirmed restored tissue architecture and minimal inflammatory infiltration in the treatment group. Moreover, these nanostructures exhibited excellent biocompatibility: negligible cytotoxicity (cell viability >90% at concentrations up to 100 µg/mL), low hemolytic activity (<5%), and no observable organ toxicity or weight loss in treated mice. Biodistribution studies further indicated efficient clearance of the nanoparticles, likely accelerated by NIR-induced hyperthermia^100^.

Dabagh et al.^125^ synthesized zeolite/CuFe_2_O_4_ nanocomposites using the co-precipitation method to enhance antibacterial activity against both Gram-positive (*Bacillus subtilis* and *Staphylococcus aureus*) and Gram-negative (*Salmonella choleraesuis* and *Escherichia coli*) bacterial strains^125^. Zeolite, a porous aluminosilicate framework containing Si, Al, and Na, serves as a structural host for CuFe_2_O_4_ nanoparticles, improving their dispersion and antibacterial performance. The nanocomposites containing 6 wt% CuFe_2_O_4_ exhibited a significantly enhanced antibacterial effect against Gram-negative bacterial strains compared to the individual zeolite and CuFe_2_O_4_. This enhancement is attributed to the synergistic action of copper and iron ions, facilitated by ion exchange within the zeolite matrix. These nanocomposites exhibited magnetic properties (*M*_*s*_ = 10–41 emu/g), allowing for easy manipulation and recovery under an external magnetic field, which supports potential reuse in biomedical and environmental applications^125^.

#### Discussion and feature perspective

##### Engineering insights from CuFe_2_O_4_-based biomedical nanosystems

Although CuFe_2_O_4_ nanoparticles have been widely studied for biomedical applications, their true significance emerges when considered as engineerable components within micro- and nanosystems, rather than as isolated materials. Among spinel ferrites, CuFe_2_O_4_ occupies a distinctive position due to its composition-dependent lattice distortion, redox activity, and moderate magnetic response. In particular, Jahn–Teller distortion induced by Cu^2+^ ions provides an additional structural degree of freedom that enables tuning of magnetic anisotropy, electronic structure, and catalytic behavior. This contrasts with other spinel ferrites: ZnFe_2_O_4_ generally forms an undistorted normal spinel; MnFe_2_O_4_ tends to a low‑anisotropy mixed spinel; and CoFe_2_O_4_/NiFe_2_O_4_ nanoparticles typically adopt inverse spinel structures with relatively minor lattice distortion^8,37^.

From a magnetic engineering perspective, CuFe_2_O_4_ offers a balanced magnetic profile characterized by moderate *M*_*s*_ and relatively low magnetic anisotropy. In contrast, CoFe_2_O_4_ and NiFe_2_O_4_ for instance, exhibit strong magnetocrystalline anisotropy, high coercivity, and significant toxicity, making them better suited for data‑storage and permanent magnet applications than for dynamically actuated biomedical systems^24,47,76^. On the other hand, ZnFe_2_O_4_ and MnFe_2_O_4_ typically display lower magnetization and coercivity due to weaker A–B superexchange, which limits their efficacy in magnetic hyperthermia or imaging^31,61,112^.

Beyond magnetism, the redox-active Cu^2+^/Cu^+^ couple introduces catalytic and chemical functionalities that distinguish CuFe_2_O_4_ from iron- and zinc-based ferrites. These properties enable reactive oxygen species generation, Fenton-like reactions, and radiosensitization, supporting synergistic therapeutic mechanisms in cancer treatment and antimicrobial applications. Cobalt-containing ferrites can also promote ROS production, but cobalt toxicity significantly limits their therapeutic safety window. In contrast, controlled Cu^2+^ release can be exploited therapeutically when appropriately engineered through particle size control and surface passivation^24,29,58,111,126^.

Recent research has expanded the biomedical scope of CuFe_2_O_4_ nanoparticles into the fields of wound healing and tissue engineering^19,127^. However, their definitive efficacy and optimal application strategies in these areas remain subjects of active investigation, requiring further dedicated exploration. In a foundational study, Liu et al.^100^ reported the in vivo treatment of infected wounds using a synergistic platform combining PTT with surface-functionalized CuFe_2_O_4_ nanoparticles^100^. In a complementary investigation, Sharifi et al.^30^ engineered a bioactive wound dressing by embedding CuFe_2_O_4_ nanoparticles within a chitosan@poly(ethylene oxide) nanofibrous scaffold^30^. Critically, both in vitro and in vivo assessments confirmed the material’s excellent biocompatibility and its efficacy in promoting skin tissue regeneration^30^. Notably, these findings highlight the benefit of embedding CuFe_2_O_4_ into polymeric or fibrous scaffolds, enabling localized antimicrobial activity and pro-regenerative effects while maintaining biocompatibility.

##### System-level considerations and clinical translation challenges

Although preclinical studies of CuFe_2_O_4_‑based nanoplatforms have shown encouraging results, their definitive therapeutic efficacy and optimal engineering parameters remain under active investigation. The successful clinical translation of these platforms depends not solely on intrinsic material properties but also on their integration into a broader system‑level framework. Critical unresolved questions encompass physiological constraints, device compatibility, and scalable manufacturing processes; all of which ultimately determine in vivo performance and safety^128^.

CuFe_2_O_4_ nanoparticles have shown promise in drug delivery, where pH-responsive drug release in acidic tumor microenvironments can be combined with copper-mediated ROS generation for enhanced anticancer efficacy^29^. This release of Cu^2+^ ion, while therapeutically useful, raises concerns regarding cumulative toxicity, oxidative stress, and long-term biosafety. Defining safe and effective dosing regimens is particularly complex, as repeated administration may exacerbate systemic toxicity or disrupt the microbiome, whereas insufficient dosing may limit therapeutic outcomes. In addition, the route and mode of administration present significant barriers. Systemic delivery is often limited by rapid clearance, off-target accumulation, and insufficient tumor penetration, whereas localized administration faces practical constraints related to accessibility, dosing uniformity, and retention at the target site. Achieving precise control over drug release kinetics in heterogeneous biological environments further complicates therapeutic predictability, as variations in pH, enzymatic activity, and tissue perfusion can alter system behavior.

In magnetic hyperthermia, CuFe_2_O_4_ nanostructures demonstrate favorable heating efficiency due to their low coercivity and superparamagnetic behavior, allowing effective temperature elevation within accepted safety thresholds. However, therapeutic performance at the system level cannot be inferred solely from intrinsic SAR values. Instead, it is determined by the interaction between nanoparticle properties, AMF parameters, tissue perfusion, and local heat dissipation. Therefore, future strategies must adopt an integrated design framework in which nanoparticle characteristics are co-optimized with field-delivery systems, including coil geometry, field homogeneity, and real-time thermal feedback, to ensure precise and spatially controlled heating under physiological conditions^129^.

While Fe_3_O_4_ serves as a clinical benchmark, CuFe_2_O_4_ nanoparticles present a compelling alternative for MRI T_2_/T_2_* contrast due to their greater compositional tunability^31^. Translating this advantage into consistent clinical performance, however, remains challenging. Major barriers include colloidal instability, aggregation in biological environments, and the difficulty of maintaining tightly controlled hydrodynamic size and surface chemistry, all of which directly affect pharmacokinetics and biodistribution. Furthermore, achieving optimal r_2_ at clinically relevant magnetic field strengths (1.5–3 T) requires careful coordination between nanoparticle design and imaging hardware. This necessitates the co-engineering of nanoparticle systems with delivery platforms (e.g., antiferromagnetic nanoprobes), alongside the optimization of AMF coil configurations and the integration of real-time thermal monitoring techniques (e.g., MRI thermometry) to achieve precise spatiotemporal control^130^.

Equally important are challenges related to scalability and standardization. The reproducible synthesis of ultrasmall, monodisperse CuFe_2_O_4_ nanoparticles remains technically demanding, particularly when transitioning to production processes compatible with good manufacturing practice (GMP). Achieving batch-to-batch consistency requires rigorous control over nucleation kinetics, growth pathways, and thermodynamic conditions. Moreover, the absence of standardized evaluation protocols for magnetic, catalytic, and biological performance limits cross-study comparability and complicates regulatory assessment.

From a translational perspective, these challenges are compounded by the need for reproducible fabrication, long-term colloidal stability, and well-defined biodistribution and clearance pathways. Addressing these limitations will require not only materials innovation but also the establishment of standardized characterization frameworks, long-term in vivo safety data, and clear regulatory pathways to enable reliable clinical validation and eventual translation.

##### Future perspectives

Future research on CuFe_2_O_4_ nanoparticles must move beyond material-centric studies toward system-level nanoengineering, in which intrinsic magnetic and catalytic properties are co-designed with device architectures, biological interfaces, and translational constraints^128^. A key paradigm shift will be the transition from isolated nanoparticle formulations to integrated functional nanosystems capable of precise actuation, sensing, and therapy within complex biological environments.

The active integration of CuFe_2_O_4_ nanoparticles into micro- and nanoscale devices represents a critical frontier for translation. Embedding these materials into MEMS-based hyperthermia applicators, implantable drug-eluting depots, or microfluidic biosensors can transform high SAR, switchable MRI contrast, and catalytic activity into feedback-controlled theranostic platforms. Achieving this integration will require addressing core engineering challenges, including microscale heat and mass transfer, signal-to-noise optimization, and long-term operational stability under physiological conditions.

One promising direction involves embedding CuFe_2_O_4_ nanoparticles within magnetically responsive lipid nanovesicles, hybrid lipid–polymer carriers, or bacteria^131^. These biomimetic platforms enable externally guided and site-specific drug delivery while enhancing circulation stability, payload protection, and controlled release. In parallel, advanced surface functionalization strategies—including conjugation with antibodies, targeting peptides, aptamers, or fluorescent reporters—can impart molecular specificity and enable multimodal imaging, such as the combination of magnetic resonance and optical tracking. The integration of stimuli-responsive layers (e.g., pH- or enzyme-sensitive coatings) further allows spatially and temporally controlled therapeutic activation within pathological microenvironments.

Beyond therapeutic delivery, nanoparticles hold substantial promise for biosensing applications due to their magnetic separability and catalytic signal amplification capabilities^91^. However, current studies remain largely proof-of-concept. Future progress requires the engineering of complete sensing systems, including integration into magnetoresistive or electrochemical interfaces, optimization of signal transduction pathways, and incorporation into lab-on-chip or microfluidic diagnostic platforms. In this context, machine-learning–assisted modeling offers a powerful tool to predict biodistribution, degradation kinetics, and ion release behavior, supporting the rational design of safer and more personalized nanomedicine.

Finally, successful clinical and industrial translation will depend on the development of scalable, green, and GMP-compatible synthesis routes, alongside standardized benchmarking protocols for CuFe_2_O_4_ nanoparticles performance. Long-term in vivo studies—particularly in metastatic cancer models—are essential to elucidate chronic toxicity, immune interactions, and clearance pathways. Through interdisciplinary collaboration spanning nanotechnology, oncology, bioinformatics, and microsystems engineering, CuFe_2_O_4_ nanoparticles are well positioned to evolve from promising nanomaterials into clinically viable components of next-generation biomedical microsystems.

## Conclusion

CuFe_2_O_4_ nanoparticles represent a compelling addition to the toolbox of materials for biomedical micro- and nanoengineered systems. Their significance lies not in any single property, but in the rare convergence of magnetic tunability, photothermal responsiveness, and redox activity within a single, structurally adaptable spinel framework. The ability of Cu^2+^-driven lattice distortions to modulate magnetic relaxation, heat dissipation, and ion release provides a level of functional programmability that is difficult to achieve with conventional ferrites. In this regard, CuFe_2_O_4_ occupies a distinct materials niche, bridging diagnostic, therapeutic, and catalytic functionalities within a unified nanoscale platform.

Realizing the full biomedical potential of CuFe_2_O_4_, however, demands a shift from material-centric optimization toward hierarchical nanoengineering. Atomic- and nanoscale control over composition, crystal symmetry, and magnetic domain structure must be complemented by deliberate surface and interface design to ensure biological compatibility, stability, and targeting capability. Equally critical is the integration of these engineered nanostructures into device- and system-level architectures, where constraints such as field safety limits, thermal transport, signal fidelity, and spatiotemporal control ultimately determine clinical relevance.

Viewed through this systems perspective, CuFe_2_O_4_ nanoparticles are best understood not as standalone agents, but as functional building blocks for next-generation theranostic platforms. Continued progress will depend on unifying materials science, micro/nanoengineering, and translational biology to address scalability, safety, and long-term performance. If these challenges are met, CuFe_2_O_4_-based nanosystems have the potential to move beyond preclinical demonstrations and contribute meaningfully to precision medicine, enabling integrated diagnostic and therapeutic strategies with real-world impact.

## Data Availability

Data of the current study are available from the corresponding author on reasonable request.

## References

[1] Corral-Nájera, K., Chauhan, G., Serna-Saldívar, S. O., Martínez-Chapa, S. O. & Aeinehvand, M. M. Polymeric and biological membranes for organ-on-a-chip devices. *Microsyst. Nanoeng.* **9**, 107 (2023). 37649779 10.1038/s41378-023-00579-z PMC10462672

[2] Wang, W. et al. Microfluidic platforms for monitoring cardiomyocyte electromechanical activity. *Microsyst. Nanoeng.* **11**, 4 (2025). 39788940 10.1038/s41378-024-00751-z PMC11718118

[3] Dobson, J. Gene therapy progress and prospects: magnetic nanoparticle-based gene delivery. *Gene Ther.* **13**, 283–287 (2006). 16462855 10.1038/sj.gt.3302720

[4] Xie, J., Liu, G., Eden, H. S., Ai, H. & Chen, X. Surface-engineered magnetic nanoparticle platforms for cancer imaging and therapy. *Acc. Chem. Res.* **44**, 883–892 (2011). 21548618 10.1021/ar200044b PMC3166427

[5] Mazgaj, R. et al. Comparative evaluation of sucrosomial iron and iron oxide nanoparticles as oral supplements in iron deficiency anemia in piglets. *Int. J. Mol. Sci.* **22**, 9930 (2021). 34576090 10.3390/ijms22189930 PMC8466487

[6] Tong, L., Zhao, M., Zhu, S. & Chen, J. Synthesis and application of superparamagnetic iron oxide nanoparticles in targeted therapy and imaging of cancer. *Front. Med.* **5**, 379–387 (2011). 22198749 10.1007/s11684-011-0162-6

[7] El Ghandoor, H., Zidan, H., Khalil, M. M. & Ismail, M. Synthesis and some physical properties of magnetite (Fe_3_O_4_) nanoparticles. *Int. J. Electrochem. Sci.* **7**, 5734–5745 (2012).

[8] Kefeni, K. K., Msagati, T. A., Nkambule, T. T. & Mamba, B. B. Spinel ferrite nanoparticles and nanocomposites for biomedical applications and their toxicity. *Mater. Sci. Eng. C.* **107**, 110314 (2020). 10.1016/j.msec.2019.110314 31761184

[9] Salih, S. J. & Mahmood, W. M. Review on magnetic spinel ferrite (MFe_2_O_4_) nanoparticles: from synthesis to application. *Heliyon* **9**, e16601 (2023). 37274649 10.1016/j.heliyon.2023.e16601 PMC10238938

[10] Mmelesi, O. K. et al. Cobalt ferrite nanoparticles and nanocomposites: photocatalytic, antimicrobial activity and toxicity in water treatment. *Mater. Sci. Semicond. Process.* **123**, 105523 (2021).

[11] Irfan Hussain, M. et al. In *Magnetic nanoheterostructures: Diagnostic, imaging and treatment* (eds Sharma, S. K. & Javed, Y.) 243–265 (Springer International Publishing, 2020).

[12] Gabal, M. A. et al. Structural and magnetoelectrical properties of MFe_2_O_4_ (M = Co, Ni, Cu, Mg, and Zn) ferrospinels synthesized via an egg-white biotemplate. *ACS Omega* **6**, 22180–22187 (2021). 34497909 10.1021/acsomega.1c02858 PMC8412908

[13] Thakur, A. & Thakur, P. (Eds.). *Soft nanoferrites for biomedical and environmental applications* (1st ed.) 348 (CRC Press, 2024).

[14] Kumari, N., Kour, S., Gurpreet, S., & Sharma, R. K. A brief review on synthesis, properties and applications of ferrites. In *AIP Conf. Proc.* **2220**, 020164 (2020).

[15] Vedrtnam, A., Kalauni, K., Dubey, S. & Kumar, A. A comprehensive study on structure, properties, synthesis and characterization of ferrites. *AIMS Mater. Sci.* **7**, 800–835 (2020).

[16] Kazemi, M. Magnetically reusable nanocatalysts in Biginelli synthesis of dihydropyrimidinones (DHPMs). *Synth. Commun.* **50**, 1409–1445 (2020).

[17] Ariffin, M. F. K. & Idris, A. Fe_2_O_3_/chitosan coated superparamagnetic nanoparticles supporting lipase enzyme from *Candida antarctica* for microwave assisted biodiesel production. *Renew. Energy* **185**, 1362–1375 (2022).

[18] Nguyen, M. T. et al. Microwave-assisted synthesis of copper ferrite nanocatalysts for synergistic plasma-Fenton degradation of Rhodamine B. *Colloids Surf. A Physicochem. Eng.* **705**, 135681 (2025).

[19] Pergal, M. V. et al. Structure and functional characteristics of novel polyurethane/ferrite nanocomposites with antioxidant properties and improved biocompatibility for vascular graft development. *Polymers* **17**, 152 (2025). 39861225 10.3390/polym17020152 PMC11768855

[20] Jasim, S. A. et al. Green synthesis of spinel copper ferrite (CuFe_2_O_4_) nanoparticles and their toxicity. *Nanotechnol. Rev.* **11**, 2483–2492 (2022).

[21] Eivazzadeh-Keihan, R. et al. Magnetic copper ferrite nanoparticles functionalized by aromatic polyamide chains for hyperthermia applications. *Langmuir* **37**, 8847–8854 (2021). 34259525 10.1021/acs.langmuir.1c01251

[22] Ansari, M. A., Baykal, A., Asiri, S. & Rehman, S. Synthesis and characterization of antibacterial activity of spinel chromium-substituted copper ferrite nanoparticles for biomedical application. *J. Inorg. Organomet. Polym. Mater.* **28**, 2316–2327 (2018).

[23] Lakshman, M. Recent developments in coupling reactions catalyzed by copper ferrite nanoparticles (CuFe_2_O_4_ NPs). *J. Synth. Chem.* **1**, 148–154 (2023).

[24] Özçelik, S. et al. Structural, magnetic, and blood compatibility studies of nickel-copper nanoferrites produced by laser ablation technique. *J. Alloys Compd.* **1042**, 184080 (2025).

[25] Kuo, S.-H. et al. Fabrication of anisotropic Cu ferrite-polymer core-shell nanoparticles for photodynamic ablation of cervical cancer cells. *Nanomaterials* **10**, 2429 (2020). 33291730 10.3390/nano10122429 PMC7761902

[26] Lachowicz, D. et al. One-step preparation of highly stable copper–zinc ferrite nanoparticles in water suitable for MRI thermometry. *Chem. Mater.* **34**, 4001–4018 (2022). 35573108 10.1021/acs.chemmater.2c00079 PMC9097161

[27] Armedya, T. P. et al. Kinetical release study of copper ferrite nanoparticle incorporated on PCL/collagen nanofiber for naproxen delivery. *Bionanoscience* **9**, 274–284 (2019).

[28] Glazkova, E. A. et al. Copper ferrite/copper oxides (I, II) nanoparticles synthesized by electric explosion of wires for high performance photocatalytic and antibacterial applications. *Mater. Sci. Eng.* **283**, 115845 (2022).

[29] Salehiabar, M. et al. Targeted CuFe_2_O_4_ hybrid nanoradiosensitizers for synchronous chemoradiotherapy. *J. Control. Release* **353**, 850–863 (2023). 36493951 10.1016/j.jconrel.2022.12.004

[30] Sharifi, E. et al. Bioactive chitosan/poly(ethyleneoxide)/CuFe_2_O_4_ nanofibers for potential wound healing. *Environ. Res.* **239**, 117448 (2023). 37858692 10.1016/j.envres.2023.117448

[31] Heydari, F. et al. Solvothermal synthesis of polyvinyl pyrrolidone encapsulated, amine-functionalized copper ferrite and its use as a magnetic resonance imaging contrast agent. *PLoS ONE* **20**, e0316221 (2025). 39913433 10.1371/journal.pone.0316221 PMC11801609

[32] Garfami, M., Jalali, A. & Salehzadeh, A. A novel CuFe_2_O_4_@ Ag nanocomposite biosynthesized by Spirulina platensis exhibits an anticancer effect on human gastric adenocarcinoma and Michigan Cancer Foundation-7 breast cancer cell lines. *Appl. Organomet. Chem.* **34**, e5971 (2020).

[33] Gu, Y. et al. Bacterial disinfection by CuFe_2_O_4_ nanoparticles enhanced by NH_2_OH: a mechanistic study. *Nanomaterials* **10**, 18 (2019). 31861627 10.3390/nano10010018 PMC7022556

[34] Pham, T. N., Huy, T. Q. & Le, A.-T. Spinel ferrite (AFe_2_O_4_)-based heterostructured designs for lithium-ion battery, environmental monitoring, and biomedical applications. *RSC Adv.* **10**, 31622–31661 (2020). 35520663 10.1039/d0ra05133k PMC9056412

[35] Abbasian, A. R., Hosseini, S. S., Shayesteh, M., Shafiee, M. & Esmaeilzaei, M. R. Ultrasonic-assisted solvothermal synthesis of self-assembled copper ferrite nanoparticles. *Int. J. Nano Dimens.* **11**, 130–144 (2020).

[36] Dippong, T., Levei, E. A. & Cadar, O. Investigation of structural, morphological and magnetic properties of MFe_2_O_4_ (M= Co, Ni, Zn, Cu, Mn) obtained by thermal decomposition. *Int. J. Mol. Sci.* **23**, 8483 (2022). 35955614 10.3390/ijms23158483 PMC9369127

[37] Dippong, T., Levei, E. A. & Cadar, O. Recent advances in synthesis and applications of MFe_2_O_4_ (M = Co, Cu, Mn, Ni, Zn) nanoparticles. *Nanomaterials* **11**, 1560 (2021). 34199310 10.3390/nano11061560 PMC8231784

[38] Akhtar, M. F. et al. A comprehensive review on the applications of ferrite nanoparticles in the diagnosis and treatment of breast cancer. *Med. Oncol.* **41**, 53 (2024). 38198041 10.1007/s12032-023-02277-2

[39] Caddeo, F., Loche, D., Casula, M. F. & Corrias, A. Evidence of a cubic iron sublattice in t-CuFe_2_O_4_ demonstrated by X-ray absorption fine structure. *Sci. Rep.* **8**, 797 (2018). 29335500 10.1038/s41598-017-19045-8 PMC5768695

[40] Marinca, T. F., Chicinaş, I. & Isnard, O. Structural and magnetic properties of the copper ferrite obtained by reactive milling and heat treatment. *Ceram. Int.* **39**, 4179–4186 (2013).

[41] Raja, A., Palasyuk, O., Schlagel, D., Lograsso, T. & Palasyuk, A. Tetragonal structure and uniaxial magnetic anisotropy in the arcmelted (Ce,Zr)_2_(Fe,M)_17_ (M = Mo, W, Co) alloys. *J. Alloy. Compd.* **1022**, 179888 (2025).

[42] Rashad, M. M., Mohamed, R. M., Ibrahim, M. A., Ismail, L. F. M. & Abdel-Aal, E. A. Magnetic and catalytic properties of cubic copper ferrite nanopowders synthesized from secondary resources. *Adv. Powder Technol.* **23**, 315–323 (2012).

[43] Ohnishi, H. & Teranishi, T. Crystal distortion in copper ferrite-chromite series. *J. Phys. Soc. Jpn.* **16**, 35–43 (1961).

[44] Masunga, N., Mamba, B. B., Getahun, Y. W., El-Gendy, A. A. & Kefeni, K. K. Synthesis of single-phase superparamagnetic copper ferrite nanoparticles using an optimized coprecipitation method. *Mater. Sci. Eng.* **272**, 115368 (2021).

[45] Masunga, N., Mmelesi, O. K., Kefeni, K. K. & Mamba, B. B. Recent advances in copper ferrite nanoparticles and nanocomposites synthesis, magnetic properties and application in water treatment: review. *J. Environ. Chem. Eng.* **7**, 103179 (2019).

[46] Balagurov, A., Bobrikov, I., Pomjakushin, V. Y., Sheptyakov, D. & Yushankhai, V. Y. Interplay between structural and magnetic phase transitions in copper ferrite studied with high-resolution neutron diffraction. *J. Magn. Magn. Mater.* **374**, 591–599 (2015).

[47] Alzoubi, G. M. The effect of Co-doping on the structural and magnetic properties of single-domain crystalline copper ferrite nanoparticles. *Magnetochemistry* **8**, 164 (2022).

[48] Yadav, R. S. et al. Cation migration-induced crystal phase transformation in copper ferrite nanoparticles and their magnetic property. *J. Supercond. Nov. Magn.* **29**, 759–769 (2016).

[49] Cobos, M. Á, Jiménez, J. A., Llorente, I., de la Presa, P. & Hernando, A. Ball milling and annealing effect in structural and magnetic properties of copper ferrite by ceramic synthesis. *J. Alloy. Compd.* **1006**, 176206 (2024).

[50] Wei, H. et al. Superparamagnetic iron oxide nanoparticles: cytotoxicity, metabolism, and cellular behavior in biomedicine applications. *Int. J. Nanomed.* **16**, 6097–6113 (2021). 10.2147/IJN.S321984 PMC8418330 34511908

[51] Nuzhina, J., Shtil, A., Prilepskii, A. & Vinogradov, V. Preclinical evaluation and clinical translation of magnetite-based nanomedicines. *J. Drug Deliv. Sci. Technol.* **54**, 101282 (2019).

[52] Salem, B., Hemeda, O., Henaish, A., Mostafa, N. & Mostafa, M. Modified copper zinc ferrite nanoparticles doped with Zr ions for hyperthermia applications. *Appl. Phys. A* **128**, 264 (2022).

[53] Yu, X., Yang, R., Wu, C., Liu, B. & Zhang, W. The heating efficiency of magnetic nanoparticles under an alternating magnetic field. *Sci. Rep.* **12**, 16055 (2022). 36163493 10.1038/s41598-022-20558-0 PMC9513098

[54] Mozhdehbakhsh Mofrad, Y., Asiaei, S., Shaygani, H. & Salehi, S. S. Investigating the effect of magnetic field and nanoparticles characteristics in the treatment of glioblastoma by magnetic hyperthermia method: an in silico study. *Results Eng.* **23**, 102473 (2024).

[55] Gavilán, H. et al. How size, shape and assembly of magnetic nanoparticles give rise to different hyperthermia scenarios. *Nanoscale* **13**, 15631–15646 (2021). 34596185 10.1039/d1nr03484g

[56] Mehdaoui, B. et al. Optimal size of nanoparticles for magnetic hyperthermia: a combined theoretical and experimental study. *Adv. Funct. Mater.* **21**, 4573–4581 (2011).

[57] Amorim, C. O. A compendium of magnetic nanoparticle essentials: a comprehensive guide for beginners and experts. *Pharmaceutics* **17**, 137 (2025). 39861783 10.3390/pharmaceutics17010137 PMC11768579

[58] Kurian, J., Lahiri, B. B., Mathew, M. J. & Philip, J. High magnetic fluid hyperthermia efficiency in copper ferrite nanoparticles prepared by solvothermal and hydrothermal methods. *J. Magn. Magn. Mater.* **538**, 168233 (2021).

[59] Asaei, S. & Norouzbeigi, R. Green agricultural wastes as templates for combustion synthesis of copper ferrite nanopowders: statistically designed assessment of the influential factors. *Ceram. Int.* **47**, 11756–11768 (2021).

[60] Abu-Elsaad, N. I. & Abdel hameed, R. E. Copper ferrite nanoparticles as nutritive supplement for cucumber plants grown under hydroponic system. *J. Plant Nutr.* **42**, 1645–1659 (2019).

[61] Martínez-Montelongo, J. H. et al. Antimicrobial activity of CuFe_2_O_4_ and CuFe_2_O_4_/ZnO: squaring colorimetric and traditional microbiology assays with atomic force- and holotomography-microscopies. *Ceram. Int.* **51**, 2452–2466 (2025).

[62] Subha, A., Shalini, M. G., Sahu, B. & Sahoo, S. C. Structural transformation and magnetic properties of copper ferrite nanoparticles prepared by sol–gel method. *J. Mater. Sci. Mater. Electron.* **29**, 20790–20799 (2018).

[63] Tang, X.-X., Manthiram, A. & Goodenough, J. Copper ferrite revisited. *J. Solid State Chem.* **79**, 250–262 (1989).

[64] Desai, M. et al. Annealing induced structural change in sputter deposited copper ferrite thin films and its impact on magnetic properties. *J. Appl. Phys.* **91**, 2220–2227 (2002).

[65] Gao, M., Ye, R., Shen, W. & Xu, B. Copper nitrate: a privileged reagent for organic synthesis. *Org. Biomol. Chem.* **16**, 2602–2618 (2018). 29565088 10.1039/C8OB00332G

[66] Ciont, C., Mesaroș, A., Pop, O. L. & Vodnar, D. C. Iron oxide nanoparticles carried by probiotics for iron absorption: A systematic review. *J. Nanobiotechnology* **21**, 124 (2023). 37038224 10.1186/s12951-023-01880-9 PMC10088223

[67] Abo Atia, T. et al. Synthesis and characterization of copper ferrite magnetic nanoparticles by hydrothermal route. *Chem. Eng. Trans.* **47**, 151–156 (2016).

[68] Zhang, W. et al. One-step facile solvothermal synthesis of copper ferrite–graphene composite as a high-performance supercapacitor material. *ACS Appl. Mater. Interfaces* **7**, 2404–2414 (2015). 25584805 10.1021/am507014w

[69] Calvo-de la Rosa, J. & Segarra, M. Optimization of the synthesis of copper ferrite nanoparticles by a polymer-assisted sol–gel method. *ACS Omega* **4**, 18289–18298 (2019). 31720529 10.1021/acsomega.9b02295 PMC6844087

[70] Altincekic, T. G. et al. Synthesis and characterization of CuFe_2_O_4_ nanorods synthesized by polyol route. *J. Alloy. Compd.* **493**, 493–498 (2010).

[71] Tajik, S. & Khodabakhshi, S. Novel and feasible synthetic routes to copper ferrite nanoparticles: Taguchi optimization and photocatalytic application. *J. Mater. Sci. Mater. Electron.* **27**, 5175–5182 (2016).

[72] Sreekala, G., Beevi, A. F., Resmi, R. & Beena, B. Removal of lead (II) ions from water using copper ferrite nanoparticles synthesized by green method. *Mater. Today. Proc.* **45**, 3986–3990 (2021).

[73] Gayathri Manju, B. & Raji, P. Green synthesis of nickel–copper mixed ferrite nanoparticles: structural, optical, magnetic, electrochemical and antibacterial studies. *J. Electron. Mater.* **48**, 7710–7720 (2019).

[74] El Messaoudi, N. et al. Green synthesis of CuFe_2_O_4_ nanoparticles from bioresource extracts and their applications in different areas: a review. *Biomass Convers. Biorefinery* **15**, 99–120 (2025).

[75] Kohli, S. et al. Template-assisted chemical vapor deposited spinel ferrite nanotubes. *J. Phys. Chem. C.* **114**, 19557–19561 (2010).

[76] Alahmari, F., Khan, F. A., Sozeri, H., Sertkol, M. & Jaremko, M. Electrospun Cu–Co ferrite nanofibers: synthesis, structure, optical and magnetic properties, and anti-cancer activity. *RSC Adv.* **14**, 7540–7550 (2024). 38440265 10.1039/d3ra08087k PMC10910578

[77] Salavati-Niasari, M. et al. Synthesis and characterization of copper ferrite nanocrystals via coprecipitation. *J. Clust. Sci.* **23**, 1003–1010 (2012).

[78] Manohar, A. et al. Evaluating Mg^2⁺^- doped Cu-Zn ferrite nanoparticles for hyperthermia and their biocompatibility in normal and cancer cells. *Ceram. Int.* **51**, 11174–11187 (2025).

[79] Ansari, M., Bigham, A., Hassanzadeh Tabrizi, S. A. & Abbastabar Ahangar, H. Copper-substituted spinel Zn-Mg ferrite nanoparticles as potential heating agents for hyperthermia. *J. Am. Ceram. Soc.* **101**, 3649–3661 (2018).

[80] Ramadan, R. & El-Masry, M. M. Effect of (Co and Zn) doping on structural, characterization and the heavy metal removal efficiency of CuFe_2_O_4_ nanoparticles. *J. Aust. Ceram. Soc.* **60**, 509–524 (2024).

[81] Haque, M. M. et al. Manganese doped copper ferrite nanoparticles: a promising approach for organic dye elimination under light irradiation. *Results Chem.* **7**, 101509 (2024).

[82] Azam, A. Microwave assisted synthesis and characterization of Co doped Cu ferrite nanoparticles. *J. Alloy. Compd.* **540**, 145–153 (2012).

[83] Singh, A., Singh, S., Singh, N. & Singh, J. Electrochemical immunosensor for C-reactive protein detection using an optimized bioinspired Ni-doped copper ferrite nanocomposite interface for cardiovascular disease management. *ACS Appl. Bio Mater.* **8**, 5078–5089 (2025). 10.1021/acsabm.5c00406 40388346

[84] Hong, Y. et al. Sol–gel synthesis of visible-light-driven Ni_(1−x)_Cu_(x)_Fe_2_O_4_ photocatalysts for degradation of tetracycline. *Ceram. Int.* **41**, 1477–1486 (2015).

[85] Dhiwahar, A. T., Sundararajan, M., Sakthivel, P., Dash, C. S. & Yuvaraj, S. Microwave-assisted combustion synthesis of pure and zinc-doped copper ferrite nanoparticles: structural, morphological, optical, vibrational, and magnetic behavior. *J. Phys. Chem. Solids* **138**, 109257 (2020).

[86] Darvish, M. et al. RETRACTED ARTICLE: biosynthesis of Zn-doped CuFe_2_O_4_ nanoparticles and their cytotoxic activity. *Sci. Rep.* **12**, 9442 (2022). 35676521 10.1038/s41598-022-13692-2 PMC9177859

[87] Hu, B. et al. Fabrication of novel rational Ti-Sn doped Cu-ferrite nanoparticles for robust photocatalysis reaction, magnetic resonance imaging, and chemo-magneto-photo-thermal therapy. *Surf. Interfaces* **33**, 102226 (2022).

[88] Jermy, B. R. et al. Targeted therapeutic effect against the breast cancer cell line MCF-7 with a CuFe_2_O_4_/silica/cisplatin nanocomposite formulation. *Beilstein J. Nanotechnol.* **10**, 2217–2228 (2019). 31807407 10.3762/bjnano.10.214 PMC6880833

[89] Hu, G. et al. Vitamin e succinate-glycol chitosan modified copper ferrite nanocomposites for lung cancer: targeted oxidative stress regulation induces cuproptosis and ferroptosis. *Chem. Eng. J.* **493**, 152408 (2024).

[90] Ahmad, J. et al. Differential cytotoxicity of copper ferrite nanoparticles in different human cells. *J. Appl Toxicol.* **36**, 1284–1293 (2016). 26918645 10.1002/jat.3299

[91] Sanko, V., Şenocak, A., Tümay, S. O. & Demirbas, E. A novel comparative study for electrochemical urea biosensor design: effect of different ferrite nanoparticles (MFe_2_O_4_, M: Cu, Co, Ni, Zn) in urease immobilized composite system. *Bioelectrochemistry* **149**, 108324 (2023). 36401962 10.1016/j.bioelechem.2022.108324

[92] Jamir, M., Borgohain, C. & Borah, J. P. Study of chitosan coated copper substituted nano-ferrites for hyperthermia applications. *Phys. E Low. Dimens. Syst. Nanostruct.* **146**, 115560 (2023).

[93] Ghazi, R. et al. Iron oxide based magnetic nanoparticles for hyperthermia, MRI and drug delivery applications: a review. *RSC Adv.* **15**, 11587–11616 (2025). 40230636 10.1039/d5ra00728c PMC11995399

[94] Khanna, P. K., Gaikwad, S., Adhyapak, P. V., Singh, N. & Marimuthu, R. Synthesis and characterization of copper nanoparticles. *Mater. Lett.* **61**, 4711–4714 (2007).

[95] Yin, Y. et al. BSA-coated copper ferrite nanoparticles loaded with doxorubicin for synergistic ferroptosis-chemotherapy for cancer treatment. *ACS Appl. Bio Mater.* **8**, 7322–7330 (2025). 10.1021/acsabm.5c00999 40685826

[96] Shao, C., Yang, J., Huang, Y. & Xing, Y. Core-shell architectures: tailoring the electromagnetic properties for enhanced absorption. *Int. J. Appl. Ceram. Technol.* **22**, e15055 (2025).

[97] Prime, R. B., Bair, H. E., Vyazovkin, S., Gallagher, P. K. & Riga, A. Thermogravimetric analysis (TGA). In *Thermal analysis of polymers: Fundamentals and applications* (eds Menczel J. D. & Prime R. B.) 241–317 (John Wiley & Sons, Inc., 2009).

[98] Rather, S.-U. et al. Morphological, structural, surface, thermal, chemical, and magnetic properties of Al-doped nanostructured copper ferrites. *Ceram. Int.* **49**, 20261–20272 (2023).

[99] Din, M. I. & Rehan, R. Synthesis, characterization, and applications of copper nanoparticles. *Anal. Lett.* **50**, 50–62 (2017).

[100] Liu, Y. et al. Multifunctional magnetic copper ferrite nanoparticles as Fenton-like reaction and near-infrared photothermal agents for synergetic antibacterial therapy. *ACS Appl. Mater. Interfaces* **11**, 31649–31660 (2019). 31407880 10.1021/acsami.9b10096

[101] Chen, N. et al. Multifunctional CuFe_2_O_4_@HA as a GSH-depleting nanoplatform for targeted photothermal/enhanced-chemodynamic synergistic therapy. *Colloids Surf. B Biointerfaces* **229**, 113445 (2023). 37441838 10.1016/j.colsurfb.2023.113445

[102] Kanagesan, S. et al. Evaluation of antioxidant and cytotoxicity activities of copper ferrite (CuFe_2_O_4_) and zinc ferrite (ZnFe_2_O_4_) nanoparticles synthesized by sol-gel self-combustion method. *Appl. Sci.* **6**, 184 (2016).

[103] Srikanth, K. & Nutalapati, V. Copper ferrite nanoparticles induced cytotoxicity and oxidative stress in Channel catfish ovary cells. *Chemosphere* **287**, 132166 (2022). 34826900 10.1016/j.chemosphere.2021.132166

[104] Meidanchi, A. & Ansari, H. Copper spinel ferrite superparamagnetic nanoparticles as a novel radiotherapy enhancer effect in cancer treatment. *J. Clust. Sci.* **32**, 657–663 (2021).

[105] Zhang, X. et al. CuFe_2_O_4_/lignin-based carbon composited photothermal and photodynamic agents for synergetic antibacterial therapy. *Int. J. Biol. Macromol.* **277**, 134206 (2024). 39069035 10.1016/j.ijbiomac.2024.134206

[106] Rajasekar, S., Farheena, M. I., Appanan, C. & Kuppusamy, S. Synthesis of magnetic copper ferrite nanosheet for highly efficient ROS generation and photothermal cancer therapy. 27 October 2025, PREPRINT (Version 1) available at Research Square, 10.21203/rs.3.rs-7778334/v1 (2025).

[107] Kamzin, A. S., Nikam, D. & Pawar, S. Study of Co_0.5_Zn_0.5_Fe_2_O_4_ nanoparticles for magnetic hyperthermia. *Phys. Solid State* **59**, 156–163 (2017).

[108] Deatsch, A. E. & Evans, E. E. Heating efficiency in magnetic nanoparticle hyperthermia. *J. Magn. Magn. Mater.* **354**, 163–172 (2014).

[109] Rivera, D. et al. Neurosurgical applications of magnetic hyperthermia therapy. *Neurosurg. Clin. N. Am.* **34**, 269–283 (2023). 36906333 10.1016/j.nec.2022.11.004 PMC10726205

[110] Tuzun, B. S. et al. Structural characterization, antioxidant and cytotoxic effects of iron nanoparticles synthesized using Asphodelus aestivus Brot. aqueous extract. *Green. Process. Synth.* **9**, 153–163 (2020).

[111] Kombaiah, K., Vijaya, J. J., Kennedy, L. J., Bououdina, M. & Al-Najar, B. Conventional and microwave combustion synthesis of optomagnetic CuFe_2_O_4_ nanoparticles for hyperthermia studies. *J. Phys. Chem. Solids* **115**, 162–171 (2018).

[112] Mohana, S. & Sumathi, S. *Agaricus bisporus* mediated synthesis of cobalt ferrite, copper ferrite and zinc ferrite nanoparticles for hyperthermia treatment and drug delivery. *J. Clust. Sci.* **35**, 129–142 (2024).

[113] Srinivas, B. T. V., Rawat, V. S., Konda, K. & Sreedhar, B. Magnetically separable copper ferrite nanoparticles-catalyzed synthesis of diaryl, alkyl/aryl sulfones from arylsulfinic acid salts and organohalides/boronic acids. *Adv. Synth. Catal.* **356**, 805–817 (2014).

[114] Chen, T.-W. et al. Facile synthesis of copper ferrite nanoparticles with chitosan composite for high-performance electrochemical sensor. *Ultrason. Sonochem.* **63**, 104902 (2020). 31951998 10.1016/j.ultsonch.2019.104902

[115] McDonald, J. S. & McDonald, R. J. MR imaging safety considerations of gadolinium-based contrast agents: gadolinium retention and nephrogenic systemic fibrosis. *Magn. Reson. Imaging Clin. N. Am.* **28**, 497–507 (2020). 33040991 10.1016/j.mric.2020.06.001

[116] Ilosvai, ÁM. et al. Development of manganese ferrite coated with Prussian blue as an efficient contrast agent for applications in magnetic resonance imaging. *Sci. Rep.* **15**, 14150 (2025). 40269150 10.1038/s41598-025-98348-7 PMC12019548

[117] Hankiewicz, J. H. et al. Zinc doped copper ferrite particles as temperature sensors for magnetic resonance imaging. *AIP Adv.* **7**, 550–557 (2017).

[118] Ghaemi, M., Tavakkoli, H., Ghaemi, A. & Kheradmand, D. Targeted antimicrobial therapy using spinel nanoparticles: combating multidrug-resistant gut pathogens while preserving commensal microbiota. *Bionanoscience* **15**, 566 (2025).

[119] Liakos, I. L. et al. Antimicrobial lemongrass essential oil—copper ferrite cellulose acetate nanocapsules. *Molecules* **21**, 520 (2016). 27104514 10.3390/molecules21040520 PMC6273162

[120] Muthukumar, K. et al. Solvothermal synthesis of magnetic copper ferrite nano sheet and its antimicrobial studies. *Mater. Chem. Phys.* **209**, 172–179 (2018).

[121] Ye, J. et al. Novel copper-containing ferrite nanoparticles exert lethality to MRSA by disrupting MRSA cell membrane permeability, depleting intracellular iron ions, and upregulating ROS levels. *Front. Microbiol*. **14**, 1023036 (2023). 10.3389/fmicb.2023.1023036 PMC9947852 36846790

[122] Hatami Kahkesh, K. et al. Synthesis, characterization, antioxidant and antibacterial activities of zinc ferrite and copper ferrite nanoparticles. *Mater. Chem. Horiz.* **2**, 49–56 (2023).

[123] Fomenko, A. N., Kondranova, A. M., Kazantsev, S. O., Lozkomoev, A. S. & Pervikov, A. V. Antimicrobial activity of CuFe_2_O_4_ nanoparticles obtained by electric explosion of Fe and Cu wires. *AIP Conf. Proc*. **2167**, 020106 (2019).

[124] Glazkova, E. et al. Antibacterial properties of PMMA functionalized with CuFe_2_O_4_/Cu_2_O/CuO nanoparticles. *Coatings* **12**, 957 (2022).

[125] Dabagh, S., Haris, S. A., Isfahani, B. K., Ashammakhi, N. & Ertas, Y. N. Zeolite-copper ferrite nanocomposites with improved antibacterial activity and reusability for biomedical applications. *ACS Appl. Nano Mater.* **6**, 21412–21423 (2023).

[126] Leyssens, L., Vinck, B., Van Der Straeten, C., Wuyts, F. & Maes, L. Cobalt toxicity in humans—a review of the potential sources and systemic health effects. *Toxicology* **387**, 43–56 (2017). 28572025 10.1016/j.tox.2017.05.015

[127] Aghajanian, A. H. et al. A 3D macroporous and magnetic Mg_2_SiO_4_-CuFe_2_O_4_ scaffold for bone tissue regeneration: surface modification, in vitro and in vivo studies. *Biomater. Adv.* **137**, 212809 (2022). 35929249 10.1016/j.bioadv.2022.212809

[128] Liu, H., Yu, H. & Li, J. Biomedical materials benefit health. *BMEMat* **1**, e12013 (2023).

[129] Ozcelik, S. Copper ferrite nanoparticles: structural, magnetic, optical, photocatalytic activity and blood compatibility properties. *BioNanoScience* **13**, 1–15 (2023).

[130] Mo, W. et al. A targeted antiferromagnetic nanoprobe for ultra-sensitive magnetic resonance imaging detection of sub-millimeter esophageal tumors. *BMEMat* **4**, e70040 (2026).

[131] Zheng, S., Li, X. & Guo, S. Nanomedicine hitchhiking on bacteria for treating tumors. *BMEMat* **2**, e12110 (2024).

[132] Naseri, M. G., Saion, E. B., Ahangar, H. A. & Shaari, A. H. Fabrication, characterization, and magnetic properties of copper ferrite nanoparticles prepared by a simple, thermal-treatment method. *Mater. Res. Bull.* **48**, 1439–1446 (2013).

[133] Ansari, M. A. et al. Sol-gel synthesis of dy-substituted Ni_(0.4)_Cu_(0.2)_Zn_(0.4)(_Fe_(2-x)_Dy_(x))_O_(4)_ nano spinel ferrites and evaluation of their antibacterial, antifungal, antibiofilm and anticancer potentialities for biomedical application. *Int J. Nanomed.* **16**, 5633–5650 (2021). 10.2147/IJN.S316471 PMC8381027 34434046

[134] Malik, M. A., Wani, M. Y. & Hashim, M. A. Microemulsion method: a novel route to synthesize organic and inorganic nanomaterials: 1st Nano Update. *Arab. J. Chem.* **5**, 397–417 (2012).

[135] Cardoso, B., Nobrega, G., Machado, M. & Lima, R. A. Green synthesis of copper ferrite-based nanofluids using *Chlorella vulgaris* for heat transfer enhancement. *J. Mol. Liq.* **428**, 127498 (2025).

[136] Yang, A., Chen, Z., Islam, S. M., Vittoria, C. & Harris, V. G. Cation engineering of Cu-ferrite films deposited by alternating target laser ablation deposition. *J. Appl. Phys*. **103**, 07E509 (2008).

[137] Manova, E. et al. Nanosized copper ferrite materials: mechanochemical synthesis and characterization. *J. Solid State Chem.* **184**, 1153–1158 (2011).

[138] Shahvelayati, A. S. & Tanha, A. A review of ionic liquids: from solvent applications to template-assisted synthesis of metal oxide nanoparticles. *Appl. Organomet. Chem.* **39**, e70026 (2025).

[139] Talaei, M., Hassanzadeh-Tabrizi, S. A. & Saffar-Teluri, A. Synthesis of mesoporous CuFe_2_O_4_@SiO_2_ core-shell nanocomposite for simultaneous drug release and hyperthermia applications. *Ceram. Int.* **47**, 30287–30297 (2021).

[140] Naik, C. C., Kamat, D. P. & Gaonkar, S. K. Assessment of the catalytic and biological potential of yttrium and samarium-modified copper ferrite nanomaterials. *Int. J. Biol. Macromol.* **268**, 131752 (2024). 38657936 10.1016/j.ijbiomac.2024.131752

[141] Havrdova, M. et al. Field emission scanning electron microscopy (FE-SEM) as an approach for nanoparticle detection inside cells. *Micron* **67**, 149–154 (2014). 25173605 10.1016/j.micron.2014.08.001

[142] Satyvaldiev, A. et al. In *IOP Conference Series: Materials Science and Engineering* 012075 (IOP Publishing).

[143] Mohamed, M. A., Jaafar, J., Ismail, A., Othman, M. & Rahman, M. in *Membrane Characterization* 3–29 (Elsevier, 2017).

[144] Dai, F., Zhuang, Q., Huang, G., Deng, H. & Zhang, X. Infrared spectrum characteristics and quantification of OH groups in coal. *ACS Omega* **8**, 17064–17076 (2023). 37214670 10.1021/acsomega.3c01336 PMC10193429

[145] Naresh, U., Kumar, R. J. & Naidu, K. C. B. Hydrothermal synthesis of barium copper ferrite nanoparticles: nanofiber formation, optical, and magnetic properties. *Mater. Chem. Phys.* **236**, 121807 (2019).

[146] Saikova, S. et al. Copper ferrite nanoparticles synthesized using anion-exchange resin: influence of synthesis parameters on the cubic phase stability. *Materials* **16**, 2318 (2023). 36984206 10.3390/ma16062318 PMC10059923

[147] Zhao, D.-L., Zeng, X.-W., Xia, Q.-S. & Tang, J.-T. Preparation and coercivity and saturation magnetization dependence of inductive heating property of Fe_3_O_4_ nanoparticles in an alternating current magnetic field for localized hyperthermia. *J. Alloy. Compd.* **469**, 215–218 (2009).

[148] Lu, H., Zheng, W. & Jiang, Q. Saturation magnetization of ferromagnetic and ferrimagnetic nanocrystals at room temperature. *J. Phys. D Appl. Phys.* **40**, 320 (2007).

[149] Faramawy, A. M. & El-Sayed, H. M. Enhancement of magnetization and optical properties of CuFe_2_O_4_/ZnFe_2_O_4_ core/shell nanostructure. *Sci. Rep.* **14**, 6935 (2024). 38521808 10.1038/s41598-024-57134-7 PMC11350087

[150] Younes, A., Kherrouba, N. & Bouamer, A. Magnetic, optical, structural and thermal properties of copper ferrite nanostructured synthesized by mechanical alloying. *Micro Nano Lett.* **16**, 251–256 (2021).

[151] Schick, C. Differential scanning calorimetry (DSC) of semicrystalline polymers. *Anal. Bioanal. Chem.* **395**, 1589–1611 (2009). 19834693 10.1007/s00216-009-3169-y

